# Comparative genomics of the miniature wasp and pest control agent *Trichogramma pretiosum*

**DOI:** 10.1186/s12915-018-0520-9

**Published:** 2018-05-18

**Authors:** Amelia R. I. Lindsey, Yogeshwar D. Kelkar, Xin Wu, Dan Sun, Ellen O. Martinson, Zhichao Yan, Paul F. Rugman-Jones, Daniel S. T. Hughes, Shwetha C. Murali, Jiaxin Qu, Shannon Dugan, Sandra L. Lee, Hsu Chao, Huyen Dinh, Yi Han, Harsha Vardhan Doddapaneni, Kim C. Worley, Donna M. Muzny, Gongyin Ye, Richard A. Gibbs, Stephen Richards, Soojin V. Yi, Richard Stouthamer, John H. Werren

**Affiliations:** 1 Department of Entomology, University of California Riverside, Riverside, California 92521 USA; 2 Present Address: Department of Biology, Indiana University, Bloomington, Indiana 47405 USA; 3 Department of Biology, University of Rochester, Rochester, New York 14627 USA; 4 School of Biological Sciences, Institute for Bioengineering and Bioscience, Georgia Institute of Technology, Atlanta, Georgia 30332 USA; 5 Present Address: Department of Entomology, University of Georgia, Athens, Georgia 30602 USA; 6 State Key Laboratory of Rice Biology & Ministry of Agriculture Key Laboratory of Agricultural Entomology, Institute of Insect Sciences, Zhejiang University, Hangzhou, 310058 China; 7 Human Genome Sequencing Center, Department of Molecular and Human Genetics, Baylor College of Medicine, Houston, Texas 77030 USA

**Keywords:** Chalcidoidea, *Wolbachia*, Comparative genomics, Parthenogenesis, Symbiosis, Biological control, Miniaturization, Methylation

## Abstract

**Background:**

Trichogrammatids are minute parasitoid wasps that develop within other insect eggs. They are less than half a millimeter long, smaller than some protozoans. The Trichogrammatidae are one of the earliest branching families of Chalcidoidea: a diverse superfamily of approximately half a million species of parasitoid wasps, proposed to have evolved from a miniaturized ancestor. *Trichogramma* are frequently used in agriculture, released as biological control agents against major moth and butterfly pests. Additionally, *Trichogramma* are well known for their symbiotic bacteria that induce asexual reproduction in infected females*.* Knowledge of the genome sequence of *Trichogramma* is a major step towards further understanding its biology and potential applications in pest control.

**Results:**

We report the 195-Mb genome sequence of *Trichogramma pretiosum* and uncover signatures of miniaturization and adaptation in *Trichogramma* and related parasitoids. Comparative analyses reveal relatively rapid evolution of proteins involved in ribosome biogenesis and function, transcriptional regulation, and ploidy regulation. Chalcids also show loss or especially rapid evolution of 285 gene clusters conserved in other Hymenoptera, including many that are involved in signal transduction and embryonic development. Comparisons between sexual and asexual lineages of *Trichogramma pretiosum* reveal that there is no strong evidence for genome degradation (e.g., gene loss) in the asexual lineage, although it does contain a lower repeat content than the sexual lineage. *Trichogramma* shows particularly rapid genome evolution compared to other hymenopterans*.* We speculate these changes reflect adaptations to miniaturization, and to life as a specialized egg parasitoid.

**Conclusions:**

The genomes of *Trichogramma* and related parasitoids are a valuable resource for future studies of these diverse and economically important insects, including explorations of parasitoid biology, symbiosis, asexuality, biological control, and the evolution of miniaturization. Understanding the molecular determinants of parasitism can also inform mass rearing of *Trichogramma* and other parasitoids for biological control.

**Electronic supplementary material:**

The online version of this article (10.1186/s12915-018-0520-9) contains supplementary material, which is available to authorized users.

## Background

*Trichogramma* (Hymenoptera: Trichogrammatidae) wasps are minute polyphagous egg parasitoids used globally for controlling a variety of agricultural insect pests [1]. They are some of the smallest known insects (smaller than the largest protozoans), with adults measuring only tenths of millimeters in length. The family Trichogrammatidae is one of the earliest branching families of the superfamily Chalcidoidea [2, 3] (henceforth referred to as chalcids). This puts *Trichogramma* in a position to help us more broadly study the evolution of chalcid parasitoids, a diverse and ecologically important insect group. Additionally, many species of *Trichogramma* contain mixtures of sexual and asexual populations, and in many, asexual reproduction is induced by the endosymbiont *Wolbachia* [4, 5]. Therefore, *Trichogramma* is an excellent group to use for investigations of the evolution, ecology, and mechanisms of asexual reproduction.

The transition to a parasitic lifestyle is often associated with reductions in genome size and complexity and the rapid evolution of particular protein families involved in host-parasite interactions [6–9]. Chalcidoidea is a derived group of hymenopteran parasitoids, estimated to contain at least half a million species [10, 11]. The superfamily has undergone rapid speciation since its emergence in the late Jurassic period [12]. Evidence points to the chalcid ancestor being a miniaturized egg parasitoid, with subsequent diversification of the superfamily resulting in lineages that evolved even more miniaturized forms (such as trichogrammatids and mymarids) and those that reverted to a larger body size but maintained many features of the miniaturized ancestor, such as highly reduced wing venation [12]. Miniaturization in insects is associated with a suite of changes, including reduction of the exo- and endoskeleton, compaction of chromatin and reduction of cell size in the nervous system, changes in allometry such as relative head and brain size, and even the loss of organs normally required for respiration and circulation in larger insects [13–17]. It is likely that these major alterations in development and morphology will be reflected in the genome of chalcids, even for those taxa that subsequently increased in size following the adaptive radiation that resulted in their amazing abundance and diversity [2, 3, 12].

*Trichogramma* are found worldwide with about 180 described species in the genus [18–20], many of which are morphologically indistinguishable from each other and exhibit complex patterns of reproductive compatibility [21–24]. Trichogrammatids have a wide host range and can parasitize insect eggs of several orders [1]. Their size varies depending on the size of the host egg and the number of eggs laid within a host, but the adult wasps are often 0.3–0.4 mm long, with body lengths under 0.2 mm not uncommon [25, 26]. The same genetic line of *Trichogramma* can produce wasps anywhere from less than 0.2 mm to greater than 0.5 mm long, indicating just how plastic their body size is [26]. This plasticity in size is also seen at the level of the cell: aminergic neuron somata in *Trichogramma evanescens* range from 1.7 μm in small adults to 4.4 μm in genetically identical large adults [27]. As a result of their small size, *Trichogramma* have a number of unique morphological features in addition to the aforementioned changes often associated with miniaturization. *Trichogramma* have the smallest known insect ommatidia (a component of the eye) [17], a large relative volume of the central nervous system, some of the largest relative measurements of encephalization found in animals (with the brain size being larger than expected for the body size and thus metabolically expensive), and a loss of larval circulatory and respiratory organs [13, 14, 16]. These reductions and simplifications make it challenging to identify them to species based on morphology.

Many populations of *Trichogramma* are asexual due to infection with parthenogenesis-inducing *Wolbachia* symbionts [4, 28, 29], and in some cases they have evolved irreversible asexuality due to the loss of sexual function, such as the ability to fertilize eggs [30, 31]. Like all other Hymenoptera, *Trichogramma* are haplodiploid, where males develop from haploid eggs, and females develop from diploid eggs. In *Trichogramma* infected with *Wolbachia*, diploidy is obtained through failed chromatid segregation during the first mitotic division of the egg [32], resulting in female offspring from unfertilized eggs, rather than from fertilization. The molecular mechanism of this failed chromatid segregation is unknown. Additionally, while diploidization is essential for producing females from unfertilized eggs, it is not sufficient. Diploid male and intersex individuals are produced in appreciable quantities, implicating the importance of epigenetic patterning for complete parthenogenesis induction [33–35], a phenomenon also described for other species of parasitoid wasps infected with parthenogenesis-inducing *Wolbachia* [36].

We present the genome sequence of one such asexual, *Wolbachia*-infected line of *Trichogramma pretiosum* and a sexual strain of the same species. We use comparative genomics to identify unique features of *Trichogramma* and chalcid wasp genomes. The asexual *Trichogramma pretiosum* genome is compared to that of the conspecific sexual line to investigate which features of the reference genome are found across the species and are not unique to the transition to asexuality. Additionally, we sequenced the methylome of the asexual *Trichogramma pretiosum* line to lay the foundation for future work on the epigenetic effects of parthenogenesis induction. The *Trichogramma pretiosum* genome provides a foundation for further studies in biological control, host-microbe interactions, and the evolution of parasitism, asexuality, and miniaturization.

## Results

### Genome statistics and completeness

The *Trichogramma pretiosum* genome assembly is a high-quality draft genome, contained in 357 scaffolds composed of 7879 contigs (Table 1). The total size of the assembly is 195,087,592 base pairs (bp), which is approximately 21 megabases (Mb) smaller than the estimated size of a close relative, *Trichogramma kaykai* [37], and 45 Mb smaller than the parasitoid *Nasonia vitripennis* [38]. The *Trichogramma pretiosum* assembly is relatively complete, with only 4.5% of arthropod marker genes found to be missing from the assembly (Table 1). A total of 12,928 genes were annotated for *Trichogramma pretiosum*, a value which is more similar to the fig wasp’s (*Ceratosolen solmsi*) repertoire of 11,412 genes than to *Nasonia vitripennis*’s 24,388 predicted genes [39]. We previously identified a near-complete *Wolbachia* genome (strain *w*Tpre) in the *Trichogramma pretiosum* assembly, which was relocated to a separate GenBank record (accession number [GenBank:LKEQ00000000]) and published separately [40]. This scaffold (scaffold 109) and associated annotations were removed from our analyses. The frequency of *k*-mers revealed that the repetitive fraction of the *Trichogramma pretiosum* genome is 30.3%. This is a lower repeat content than that for *Nasonia vitripennis* (40%), but much higher than that for *Apis mellifera* (10%), which is especially repeat-sparse. Details on repetitive elements and other additional analyses including immunity genes are provided in Additional file 1: Section S6 [41–45].

**Table 1:** Genome assembly statistics for an asexual line of *Trichogramma pretiosum*

Statistic	*Trichogramma pretiosum*
Scaffolds	357
Total length of scaffolds	195,087,592
Total ungapped length	180,028,424
Scaffold N50	3,706,225
Contigs	7879
Contig N50	78,655
Predicted genes	12,928
BUSCO score^a^	C:91.8%[D:11.6%],F:3.5%,M:4.5%,n:2675

^a^Benchmarking Universal Single-Copy Orthologs (BUSCO) score in standard BUSCO notation (*C* complete, *D* duplicated, *F* fragmented, *M* missing, *n* number of genes used)

### Comparative genomics

**Fig. 1:**
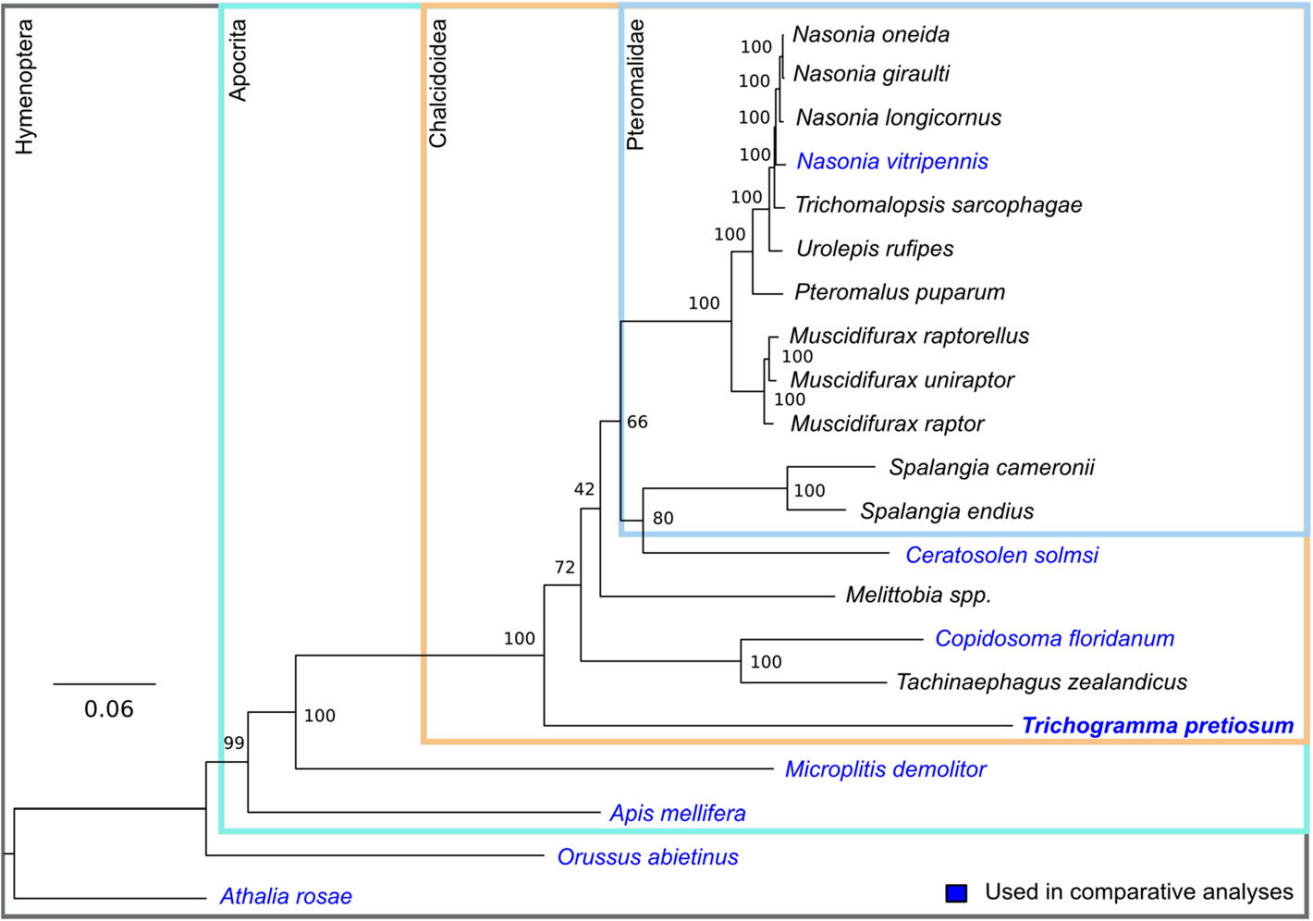
Hymenopteran phylogeny. Taxa in *blue* were used in further comparative analyses. *Trichogramma pretiosum* is in *boldface*. Bootstrap values are shown at each node. Major clades of interest are shown in *boxes*. Apocrita is a suborder that is distinguished from the earlier branching symphytans (sawflies) by their narrow “wasp-waist.” The ancestral state of Apocrita is thought to be that of parasitism, and the narrow waist an adaptation to that lifestyle. Within chalcids the majority of genomic data available are for species in the Pteromalidae, which is a large polyphyletic group of parasitoids

**Fig. 2:**
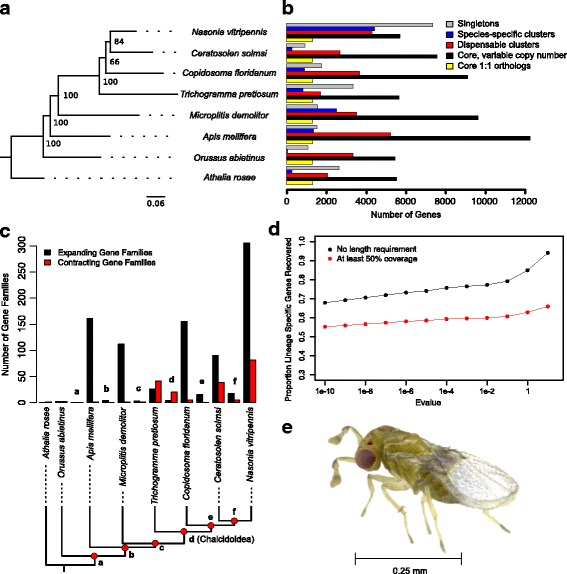
*Trichogramma pretiosum* comparative genomics. **a** Trimmed phylogeny representing relationships of species used in genomic comparisons. **b** For each species, we determined the number of genes in the single-copy core clusters, variable-copy number core clusters, dispensable clusters (non-core, non-species specific), species-specific clusters, and singleton categories, as delineated by OrthoMCL. **c** Numbers of gene family clusters that have significantly expanded or contracted at branches across the phylogeny. *Lowercase letters above pairs of bars* refer to branches leading to internal nodes of the tree and corresponding points on the phylogeny in *red*. **d** Proportion of *Trichogramma pretiosum* lineage-specific genes (delimited by OrthoMCL) recovered in other hymenopteran genomes, using blastp and a range of e-value and length thresholds. **e** Female *Trichogramma pretiosum* from the asexual Insectary line. Her body is approximately three tenths of a millimeter long

Phylogenetic reconstruction of 21 hymenopteran species recapitulated results from studies with much more substantial taxonomic sampling (Fig. 1) [46, 47]. The phylogeny places the family Trichogrammatidae as sister to the other chalcids that currently have genome sequences, although tiny egg parasitoids in the family Mymaridae (“fairyflies”) are thought to be the earliest branching chalcid lineage [12]. For genomic comparisons, the phylogeny was trimmed to eight species: four chalcids (*Nasonia vitripennis*, *Copidosoma floridanum*, *Ceratosolen solmsi*, and *Trichogramma pretiosum*), the parasitoid *Microplitis demolitor* (not a chalcid, but still within Apocrita), the honey bee *Apis mellifera* (a non-parasitoid apocritan), the parasitic wood wasp *Orussus abietinus* (from the earliest branching parasitic lineage, sister to Apocrita), and the turnip sawfly *Athalia rosae* (from the earliest branching hymenopteran family, not parasitic) (Table 2, Fig. 2a). Across the eight hymenopteran genomes, we identified 14,168 gene family clusters, using OrthoMCL [48]. On average, gene family clusters were composed of 7.6 genes. The largest gene family had 187 genes, and the second largest gene family contained 175 genes, 134 of them belonging to *Apis.*

**Table 2:** Species used in comparative analyses

Species	Common name	Family	Superfamily	Assembly	Body Size
*Apis* *mellifera*	European honey bee	Apidae	Apoidea	AADG00000000	~ 10 mm [126]
*Athalia* *rosae*	Sawfly	Tenthredinidae	Tenthredinoidea	AOFN00000000	~ 7 mm [127]
*Ceratosolen solmsi*	Fig wasp	Agaonidae	Chalcidoidea	ATAC00000000	~ 3 mm [105]
*Copidosoma floridanum*	Polyembryonic wasp	Encyrtidae	Chalcidoidea	JBOX00000000	~ 1 mm [128]
*Microplitis demolitor*	Braconid parasitoid wasp	Braconidae	Ichneumonoidea	AZMT00000000	~ 4 mm [129]
*Nasonia vitripennis*	Jewel wasp	Pteromalidae	Chalcidoidea	AAZX00000000	~ 2 mm [130]
*Orussus abietinus*	Parasitic wood wasp	Orussidae	Orussoidea	AZGP00000000	~ 11 mm [131]
*Trichogramma pretiosum*	*Trichogramma* wasp	Trichogrammatidae	Chalcidoidea	JARR00000000	~ 0.3 mm [26]

Using the results from OrthoMCL clustering, we estimated the number of genes in the single-copy core clusters, variable-copy number core clusters, dispensable genome clusters (present in two to seven species), species-specific clusters, and singleton genes for each taxon (Fig. 2b). The core, single-copy set of orthologs was 1311 clusters for Hymenoptera (yellow bars in Fig. 2b) and 3492 gene clusters for the chalcids, 100 of which were unique to the chalcid clade. When copy number was not considered, the core gene set of all the hymenopteran species was composed of 5204 gene clusters, with 6382 clusters for chalcids, 159 of which were unique to chalcids. Chalcid-specific gene family clusters were overrepresented for 12 Gene Ontology (GO) terms, including functions related to cellular organization as well as perception of and response to stimuli, which may relate to the evolution of smaller size and potentially host-seeking behaviors (see Additional file 2: Table S13).

There were 285 gene clusters not detected in any of the chalcids (*Trichogramma*, *Nasonia*, *Ceratosolen*, and *Copidosoma*) but found in all the other hymenopterans. These gene families are significantly overrepresented for 12 GO terms, eight of them related to signal transduction (see Additional file 2: Table S13). The missing (or especially divergent) chalcid genes include those for key proteins and transcription factors relating to the control of development, embryo segmentation, and dorsal-ventral patterning including putative homologs of *sog* (a growth factor), krueppel-1 (a mediator of juvenile hormone signaling), knirps (involved in segmentation), protein winged eye (determination of imaginal disc identity), and a homeobox-like protein. Several of these genes had already been identified as missing in *Nasonia* but important for dorsal-ventral patterning in *Drosophila* [49]. These include enhancer of split, trithorax, a centaurin-like protein, and F-box and leucine-rich repeat protein 7 [49]. All extant lineages of chalcids have maintained features of ancestral miniaturization (e.g., highly reduced wing venation) [12], and they are likely to show genomic signatures of this ancestral state. Indeed, developmental studies of *Nasonia* embryos revealed novel genetic and molecular developmental networks [49], consistent with idea of a novel developmental plan ancestral to Chalcidoidea [49]. Our data suggest that these may be a feature of chalcid evolution more broadly, and that these shared chalcid-unique and missing gene families may underlie the adaptive radiation of wasps in the superfamily.

### Species-specific and missing enes in *Trichogramma pretiosum*

Total numbers of species-specific genes (singletons in addition to species-specific clusters) and missing gene clusters estimated by the original OrthoMCL analysis are summarized in Table 3. *Trichogramma pretiosum* has the largest number of estimated missing clusters and the second largest number of estimated species-specific genes, as compared to the other species. However, we found through additional blast searches that many of the “missing genes” are rapidly evolving orthologs of genes in other species, and that OrthoMCL has overestimated the number of species-specific genes in *Trichogramma*.

**Table 3:** Total numbers of species-specific genes and missing genes for each genome

Genome	Species-specific genes^a,c^	Missing gene families^b,c^
*Athalia rosae*	2933	267
*Orussus abietinus*	1148	139
*Apis mellifera*	2932	172
*Microplitis demolitor*	4085	169
*Trichogramma pretiosum*	4203 (1090)^d^	403 (48)^d^
*Copidosoma floridanum*	2704	125
*Ceratosolen solmsi*	1225	110
*Nasonia vitripennis*	11,809	51

^a^Species-specific genes include singletons and genes within species-specific clusters

^b^Numbers of gene family clusters for which the species in question is the only species to not have at least one representative gene for that family

^c^Designation of “missing” and “specific” categories is as determined by OrthoMCL

^d^Corrected counts of species-specific and missing genes following manual curation in *Trichogramma pretiosum* (see section on “Species-specific and missing genes in *Trichogramma pretiosum*”)

We investigated whether or not the apparent 4203 *Trichogramma pretiosum* “species-specific genes” were truly species-specific or divergent enough to fall below the clustering threshold for orthology detection. Indeed, blastp results show that even with a stringent e-value threshold of 1e-10 and the requirement that the *Trichogramma pretiosum* gene cover at least 50% of the length of the protein in another genome, more than half (55%, *n* = 2320) of the species-specific genes are recovered in the other hymenopteran species (Fig. 2d). With an e-value threshold of 1e-5 and no length requirement, 74% (*n* = 3113) of the “species-specific” *Trichogramma pretiosum* genes are recovered in other hymenopterans, indicating that most of these genes are not truly species-specific, but likely rapidly evolving such that they fall below the original orthology detection threshold. *Trichogramma* proteins did not cluster with the other hymenopteran proteins in the OrthoMCL analysis because of either (1) having identities lower than 70% as compared to the other hymenopteran proteins (the identify threshold used in clustering) or (2) not clustering tightly enough with the homologs (the inflation parameter). These recovered genes that were significantly diverged from their hymenopteran homologs represent a quarter (24%) of the entire set of *Trichogramma pretiosum* coding sequences. While it is well established that there is a tradeoff between the ability to detect divergent orthologs and incorrectly clustering out-paralogs in orthology analyses [50], it is notable that such a large proportion of the *Trichogramma* coding sequences failed to cluster with other hymenopteran proteins. Furthermore, it is interesting that the proteins in this category are related to DNA packaging, nucleosome assembly, and chromatin (see Additional file 3: Table S14): a possible connection to the unique chromatin compaction seen in minute insects like *Trichogramma* [13, 14]. After correcting for the identification of 3113 “species-specific” *Trichogramma* proteins in other hymenopteran genomes, only 1090 of the *Trichogramma* proteins retain their species-specific status. These “true” species-specific genes are overrepresented for a number of GO terms including metabolic processes, the regulation of transcription, and signaling (see Additional file 3: Table S14).

We also assessed the status of the large number (*n* = 403) of apparent *Trichogramma*-specific “missing genes” (Table 3). We investigated whether or not they were truly absent, or if they were divergent or pseudogenized. *Nasonia vitripennis* representatives for each of these gene families were used to tblastn search for these “missing genes” in the genome sequence of *Trichogramma pretiosum*. Using an e-value cutoff of 1e-10, 355 of these genes were recovered in *Trichogramma*. Furthermore, 311 out of the 355 recovered apparent “missing genes” are hits to annotated genes. It appears that most of these are not missing, but instead were not initially assigned to gene families because of rapid evolution relative to that of the outgroups. Cross-referencing these 311 genes with the list of *Trichogramma* singletons revealed that 254 of the “missing genes” had been assigned singleton status, thus inflating our counts both for missing genes and singleton genes. Adjusted counts of lineage-specific and missing genes are shown in parentheses in Table 3. However, note that manual curation of lineage-specific and missing genes was not performed for the other species. Regardless, *Trichogramma pretiosum* appears to be an outlier with regard to the numbers of proteins that fail to cluster with the other hymenopteran proteins, while the chalcids as a group are characterized by a separate set of missing or especially divergent genes.

### Gene family expansions and contractions

The numbers of gene families that have significantly expanded or contracted along branches in the phylogeny are represented in Fig. 2c. As expected based on the number of genes in species-specific clusters and in the core genome, *Nasonia vitripennis* and *Apis mellifera* had the highest numbers of significantly expanded gene families (306 and 161 families, respectively). There is a clear trend of increased numbers of gene family contractions in Chalcidoidea, both on the branch leading to Chalcidoidea and along each lineage within Chalcidoidea. There are very few lineage-specific contractions in the branches leading to *Microplitis demolitor* and *Orussus abietinus*, implying that these contractions are a unique feature of chalcid evolution and not necessarily associated with parasitic wasps in general. While the chalcids do show higher numbers of contracting gene families, there is still a significant amount of species-specific gene family expansion that has occurred. The only branches of the phylogeny that experienced more contractions than expansions were those leading to the Chalcidoidea (expansions = 4, contractions = 20) and to *Trichogramma pretiosum* (expansions = 26, contractions = 41). *Nasonia vitripennis* and *Trichogramma pretiosum* had the highest number of unique genes (Table 3), but *Trichogramma pretiosum* had relatively few significantly expanding gene families due to its species-specific genes primarily being singletons as opposed to occurring in species-specific clusters.

### Protein evolution in *Trichogramma*

Phylogenetic reconstruction of the Hymenoptera (Fig. 1, based on 107 single-copy protein-coding genes) revealed an especially long branch leading to *Trichogramma pretiosum*. We looked across a larger set of single-copy orthologs to determine whether or not *Trichogramma* proteins are evolving faster in particular functional categories, or if sequences are evolving faster across the genome. To do so, we defined a set of core genes (*n* = 3180) that were present as single-copy genes in *Apis mellifera, Nasonia vitripennis*, and *Trichogramma pretiosum* for use in a series of comparative analyses, using *Apis* as the outgroup. Tajima’s relative rate tests followed by overrepresentation analyses and correction for multiple comparisons revealed a suite of GO terms associated with more rapidly evolving genes. Masking low-quality regions of the alignments had little effect on the GO terms overrepresented, but as expected, decreased the total number of genes found to have significantly different rates of evolution. Without masking the alignments, 1023 genes had evidence for elevated rates of protein evolution: 832 in *Trichogramma* and 191 in *Nasonia*. After masking the alignments, 590 genes showed elevated rates of protein evolution: 441 in *Trichogramma* and 149 in *Nasonia*. Thus, *Trichogramma* appears to be characterized by an enriched set of rapidly evolving genes.

In contrast to *Nasonia*, which has no overrepresented GO terms in its more rapidly evolving gene set, *Trichogramma* shows clear GO term enrichments. In *Trichogramma*, 30 GO terms were overrepresented, with 23 of them overrepresented in both masked and unmasked versions of the analyses (see Additional file 4: Table S15). Significantly overrepresented GO terms include those relating to signaling and regulatory processes, specifically regulation of nucleotide metabolism and transcription. By concatenating the alignments and performing the same relative rate test, we find that the *Trichogramma* branch is indeed significantly longer overall (*p* < 0.0001). This lends further support to the rapid evolution along the *Trichogramma* branch. Although the causes of this remain unclear, we speculate that it could have to do with the short generation time of these wasps (approximately 10 days per generation in the laboratory), the unusual feeding ecology (both larvae and adults primarily utilize the eggs of moths and butterflies), and/or the consequences of adaptation to the very small size of insects in this lineage.

### Protein evolution across Hymenoptera and Chalcidoidea

We took an additional approach to assess protein evolution rates across Hymenoptera to ensure that the identification of rapidly evolving proteins in *Trichogramma* was not due to the fact that comparisons were only made to *Nasonia*. Due to the long evolutionary times and complications of saturation in synonymous substitutions, we did not utilize nonsynonymous divergence (dN)/synonymous divergence (dS), but rather relative rates of protein evolution as a metric. Here, we reconstructed the phylogeny of each of the core single-copy hymenopteran proteins (*n* = 1311) and extracted the branch lengths for each protein from each species to the root of a clade (to Chalcidoidea for comparisons among the superfamily or to Hymenoptera for comparisons across the order). Then, branch lengths were scored within each orthologous group (with 1st place being the longest branch), allowing us to identify which ortholog had the most amino acid substitutions after divergence from the common ancestor. Within chalcids, a protein was significantly more likely to have the longest branch length to *Trichogramma* (Table 4; chi-squared statistic: *p* < 0.0001). In 580 of the 1311 single-copy protein phylogenetic reconstructions, the branch to *Trichogramma* was the longest of all chalcids. By chance, only 328 of the phylogenetic reconstructions would have the *Trichogramma* ortholog as the longest branch. There was no significant difference in the number of 1st place proteins between *Copidosoma* and *Ceratosolen* (chi-squared statistic: *p* = 0.6263). In contrast, *Nasonia* had significantly fewer proteins with the longest branch of the orthologous group, compared to the other chalcids (chi-squared statistic: *p* < 0.0001). This establishes that *Trichogramma* has more rapidly evolving proteins than do the other sequenced chalcids in this analysis. Across all Hymenoptera (with the sawfly *Athalia* as outgroup), *Trichogramma* was also significantly more likely to have the ortholog with the longest branch length (Table 5; chi-squared statistic: *p* < 0.0001). *Trichogramma* had 490 1st place proteins. *Copidosoma*, which has the next highest number of 1st place proteins, only has 290. This establishes that rates of *Trichogramma* protein evolution are higher than those for other hymenopterans examined here.

**Table 4:** Numbers of chalcid proteins with 1^st^–4th longest branch lengths

Place^a^	*Trichogramma pretiosum*	*Copidosoma floridanum*	*Ceratosolen solmsi*	*Nasonia vitripennis*
1st	580	353	341	37
2nd	344	509	339	123
3rd	263	335	398	315
4th	124	114	233	836

^a^1^st^ place means a protein had the longest branch length within an orthologous group, whereas 4th place is the shortest branch length. Placements are based on branch length from the chalcid ancestor to each chalcid species for the 1311 core, single-copy hymenopteran proteins

**Table 5:** Numbers of hymenopteran proteins with 1st–7th longest branch lengths

Place^a^	*Trichogramma pretiosum*	*Copidosoma floridanum*	*Ceratosolen solmsi*	*Nasonia vitripennis*	*Microplitis demolitor*	*Apis mellifera*	*Orussus abietinus*
1st	490	290	274	33	162	54	8
2nd	311	402	266	78	160	66	29
3rd	231	318	313	187	174	64	24
4th	148	181	294	381	189	84	39
5th	87	81	123	364	391	171	88
6th	34	30	39	207	179	538	284
7th	10	9	2	61	56	334	839

^a^1^st^ place means a protein had the longest branch length within an orthologous group, whereas 7th place is the shortest branch length. Placements are based on branch length from the hymenopteran ancestor to each species for the 1311 core, single-copy hymenopteran proteins. *Athalia rosae* was excluded due to its position as the sister to the other Hymenoptera in our analyses

Proteins that had the longest branch from the chalcid ancestor to *Trichogramma* were significantly enriched for 19 GO terms, including nucleotide metabolic processes, transcription, and the regulation of gene expression (see Additional file 5: Table S16). There was no significant enrichment of any GO terms in the set of proteins where the branch to *Trichogramma* was the shortest of the orthologous group. Neither *Nasonia* nor *Copidosoma* had GO term enrichments for proteins in which these species represented either the longest or shortest branch. In contrast, orthologous groups in which the longest branch led to *Ceratosolen*, the fig wasp, were significantly overrepresented for mitochondrial functions such as ATP production and oxidation-reduction processes (see Additional file 5: Table S16). Lastly, proteins with 1st place assigned to *Apis* were overrepresented for GO terms associated with organic acid metabolism, likely a feature of adaptation to processing nectar and pollen.

**Fig. 3:**
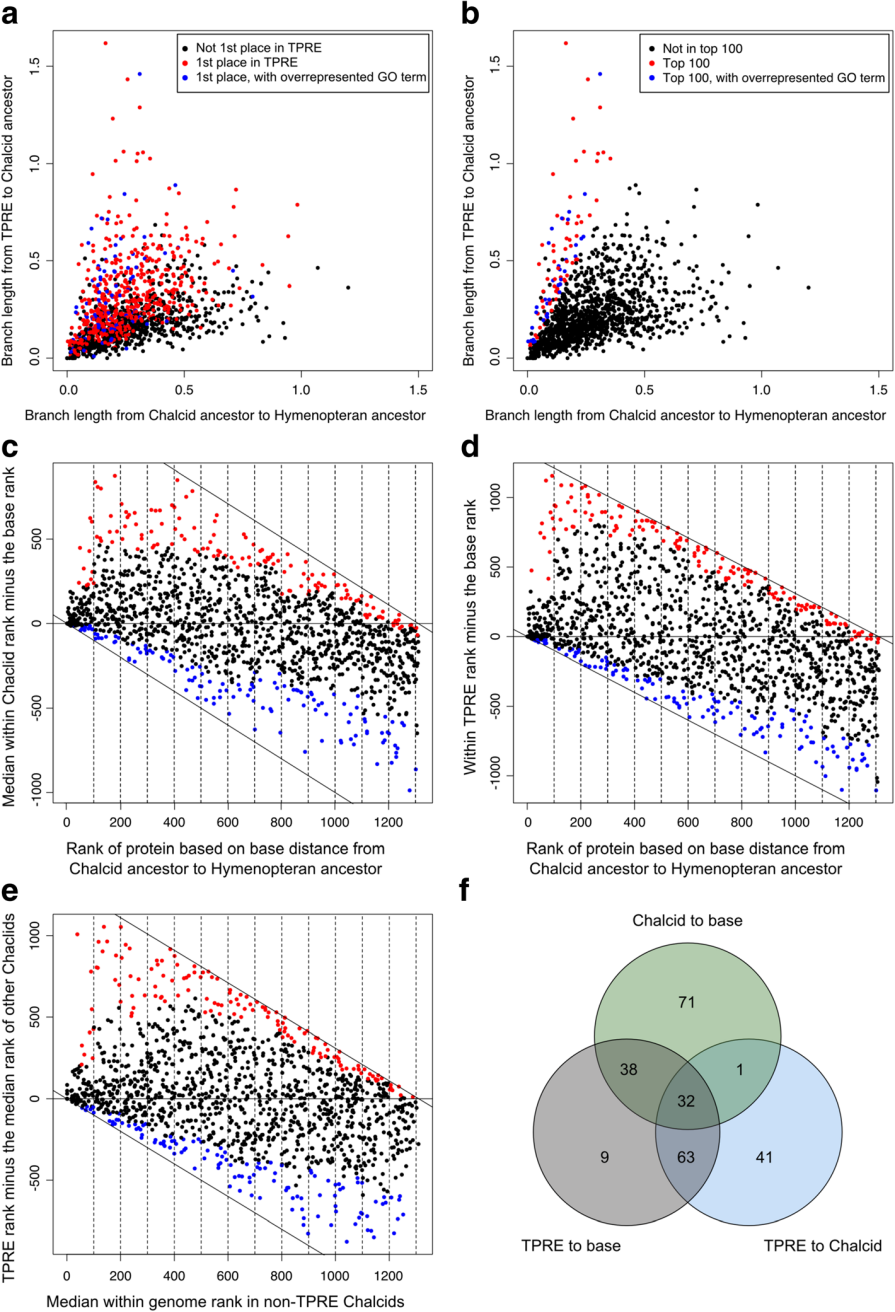
Evolution of core hymenopteran proteins. Branch lengths of proteins from *Trichogramma* to the chalcid ancestor, relative to the branch length from the chalcid ancestor to the hymenopteran ancestor highlighting (**a**) proteins with the longest branch leading to *Trichogramma* (*TPRE*) as compared to other chalcids (1st place) and (**b**) 1st place proteins with the 100 longest branch lengths after normalization. In each panel, *blue dots* represent proteins that are contributing to the overrepresentation of GO terms within either the 1st place (**a**) or longest 100 of the 1st place sets of proteins (**b**). The differences in within-genome rankings of the chalcids compared to ranks of proteins based on their base distance (from the chalcid ancestor to the hymenopteran ancestor) (**c**), *Trichogramma* within-genome rankings compared to the base rank (**d**), and *Trichogramma* within-genome rankings compared to the median within-genome ranking of the other three chalcids (**e**). In **c**, **d**, and **e**, higher values on the *x*-axis indicate a longer branch length relative to other proteins. Positive values on the *y*-axis indicate an increase in rank for the clade in question, indicating a relatively longer branch length. *Red dots* indicate the top 10% of proteins, per bin, with the largest positive discrepancy in within-genome rank. *Blue dots* indicate the bottom 10% of proteins, per bin, with the largest negative discrepancy in within-genome rank. **f** The overlaps of the “fast” proteins (*red dots*) in panels **c**, **d**, and **e**

Proteins differ in their baseline rates of evolution [51, 52]. Therefore, to identify *Trichogramma* proteins that had longer branch lengths than expected for that orthologous group, we corrected for the background rate of evolution by normalizing the “chalcid-to-species” branch lengths using the distance from the chalcid common ancestor to the hymenopteran common ancestor. In *Trichogramma*, proteins with the 100 longest normalized branch lengths (within the 1st place protein set) were significantly overrepresented for 24 GO terms, again largely related to nucleotide metabolism, transcription, and gene expression (Fig. 3a; see Additional file 5: Table S16). These overrepresented GO terms belonged to proteins with a range of “chalcid-to-Hymenoptera” branch lengths, indicating that this set of 100 more rapidly evolving *Trichogramma* genes are not just proteins subject to more relaxed selection in general (Fig. 3b). The GO terms that are significantly overrepresented in the 1st place and top 100 longest categories are the same as many of the aforementioned GO terms identified in the singleton and rapidly evolving (as determined by Tajima’s relative rate) categories, in agreement with our previous results.

Protein branch lengths significantly correlated between genomes (Spearman’s rank: *p* < 0.0001), confirming that more rapidly evolving proteins in one genome were likely to be rapidly evolving in other genomes [51, 52]. To determine which proteins had faster or slower rates of evolution across chalcids as a whole and in *Trichogramma* specifically, we first ordered and ranked the set of proteins (from 1 to 1311) within each chalcid species by their “chalcid-to-species” branch length: the within-genome rank. Within-genome ranks were significantly correlated between genomes (Spearman’s rank: *p* < 0.0001). Then, we assigned ranks to proteins based on their distance from the chalcid ancestor to the hymenopteran ancestor, i.e., the base rank. By comparing the median within-chalcid species rank to the base rank, we identified proteins for which the rank changed after the divergence from the chalcid ancestor (Fig. 3c). For example, a protein with a base rank of 10 (indicating a relatively short distance from the chalcid ancestor to the hymenopteran ancestor compared to other proteins) and a median within-chalcid rank of 1000 (indicating a relatively long branch from the chalcid ancestor to the species, compared to other proteins) would receive a score of + 990. To avoid overrepresentation among any protein category based on basal evolutionary rates, proteins were binned into groups of 100 based on the base rank (Fig. 3c). For example, bin “1–100” contains the 100 proteins with the shortest chalcid-to-Hymenoptera ancestor distances, and within each bin we selected the proteins with the largest discrepancy (top 10% and bottom 10%) in rank between the chalcid median rank and the base rank (Fig. 3c). We then used the same method to look specifically for *Trichogramma* genes with unusually ranked proteins, this time comparing the within-*Trichogramma* rank of the protein to the base rank of the protein (Fig. 3d). Lastly, to identify proteins evolving differently in *Trichogramma* relative to the other chalcids, we compared the within-*Trichogramma* rankings to the median rank of the other chalcids (*Copidosoma*, *Ceratosolen*, and *Nasonia*) instead of the base rank (Fig. 3e). The results of these three different comparisons were “fast” and “slow” proteins in each of the three categories: (1) chalcids to the base (Fig. 3c), (2) *Trichogramma* to the base (Fig. 3d), and (3) *Trichogramma* to the other chalcids (Fig. 3e). For each of these lists of proteins, we looked for overrepresented GO terms. Protein and GO term lists for each category are provided in Additional file 6: Table S17.

For proteins more rapidly evolving in chalcids, there was significant overrepresentation of GO terms associated with ribosomes and programmed cell death. Manual annotation revealed that the fastest evolving proteins in chalcids (relative to the base rank) are enriched for genes with inferred functions in cell size, proliferation, and ploidy. It is noteworthy that cell ploidy level and endoduplication can also affect cell size [53]. Examples include Nuak family SNF1 kinase (5th fastest, involved in cell ploidy), LTV1 (6th, in cell size and endoduplication), Zinc Finger FYVE protein 6 (7th, in cell adhesion), Transcription Factor AR1 (13th, in cell proliferation and apoptosis), and escargot-like (23rd, in maintenance of diploidy). We therefore argue that rapid evolution of these proteins could be involved in the proposed miniaturization of the chalcid ancestor [12]. There were no overrepresented GO terms for “slow” chalcid proteins. These findings suggest that ribosome structure and cell ploidy regulation could be a fruitful topic for study in chalcids.

Fast *Trichogramma* proteins (as compared to the base and to other chalcids) were again overrepresented for GO terms related to gene expression, nucleotide metabolism, and transcriptional processes. Manual annotation of the fastest evolving *Trichogramma* proteins relative to other chalcids (based on rank change) indicate genes involved in head structure and cell proliferation, including goosecoid homeobox (5th fastest), ETS-related transcription factor Elf-5 (6th, expressed in epithelial cells), forkhead domain containing gene crocodile-like (7th), the pair-rule and bristle formation gene hairy (9th), and transmembrane protein 45B (11th, implicated in cell proliferation). Relatively slow evolving *Trichogramma* proteins (compared to the base rate) were overrepresented for processes associated with actin and cytoskeleton organization. In contrast, slow *Trichogramma* proteins, as compared to other chalcids, were overrepresented for mitochondrial-interacting GO terms: oxidation-reduction, generation of precursor metabolites and energy, oxidative phosphorylation, and electron transport chain. This is especially interesting, as these are categories of genes that had previously been identified as more rapidly evolving in other parasitoids, such as *Nasonia* [38]. In the *Nasonia* complex, nuclear-mitochondrial incompatibilities figure prominently in reproductive isolation between closely related species [38, 54, 55] and show accelerated rates of mitochondrial evolution [56]. In contrast, *Trichogramma* spp. have unusual and difficult-to-predict patterns of intra- and interspecific reproductive compatibilities [21–24], which may be explained by the relatively slower evolution of nuclear genes interacting with mitochondria.

Lastly, we looked at the overlap of the “fast” proteins in all three categories (Fig. 3f). The 71 proteins that are unique to the “chalcid-to-base” only category include a suite of ribosomal biogenesis and ribosomal proteins, suggesting extensive evolution of the ribosomal complex and transcriptional machinery specific to the origin of chalcids. There are only nine proteins in the *Trichogramma*-to-base only category, but they include four proteins associated with polymerase I, II, or III. The 32 proteins present in the overlap of all three categories include several proteins related to maintenance or determination of ploidy and cell cycle regulation, potentially relating to cell size [53], so the evolution of these genes may be relevant to the evolution of miniaturization in the chalcid ancestor and further size reductions in *Trichogramma*. In sum, we find strong evidence for a rapid rate of protein evolution in *Trichogramma pretiosum*, and more generally in chalcids, that is particularly evident in categories relating to the regulation of gene expression and transcription, development, and ploidy. These may be involved in selection for size miniaturization and adaptation to a life as a tiny, quickly developing egg parasitoid.

### Comparison to a sexual *Trichogramma pretiosum*

The reference asexual line of *Trichogramma pretiosum* represents a relatively recent transition to asexuality. Sexual function has been lost to the point where *Wolbachia* is essential for maintenance of the colony, but females are able to mate and fertilize eggs at a low level [57]. Nevertheless, we wanted to determine whether or not features of the *Trichogramma pretiosum* reference genome were representative of the *Trichogramma pretiosum* clade as a whole, or the fact that the sequenced line is asexual, and may have undergone gene loss or divergence due to asexuality. We therefore assembled a short insert draft genome (using paired-end 250-bp reads) for a sexually reproducing line of *Trichogramma pretiosum*, “CA-29,” which is naturally uninfected with *Wolbachia* (see Additional file 1: Section S4) [19, 57–68]. The sexual line is derived from a population in California, whereas the asexual line is from a population in Peru. We obtained approximately 70× coverage of the CA-29 *Trichogramma* draft genome, resulting in a de novo assembly of 189,939,323 bp into 40,567 contigs. Clearly the quality of this assembly is inferior to the reference asexual line. Nevertheless, it can be used to address some key questions, such as whether or not features of the reference genome are a result of the transition to asexuality or are conserved features of *Trichogramma pretiosum* independent of reproductive type.

Depending on circumstances, models predict either an increase or decrease in the abundance of repetitive DNA as a consequence of the transition to asexual reproduction [69–73]. We therefore compared the repetitive elements present in the sexual and asexual *Trichogramma* genomes using *k*-mer-based approaches (see Additional file 1: Section S6) [41–44, 74]. The size of the sexual genome as predicted by *k*-mer frequencies was 193.9 mega base pairs (Mbp), with a repetitive fraction of 26.2%. This indicates that there was no significant reduction in the size or repeat content of the asexual genome (195 Mbp and 30.3%, respectively), as compared to the sexual one. However, within that total repetitive fraction the sexual and asexual genomes had different proportions of the types of repetitive elements. We constructed repeat libraries containing transposons and tandem repeats and mapped genomic reads back to these sequences to estimate their abundance in both genomes. The sexual genome had a significantly higher percentage of reads mapping to transposons and tandem repeats, as compared to the asexual genome (~ 12% and ~ 8%, respectively, chi-squared statistic: *p* < 0.001), and had a greater abundance of some specific transposon-like elements and tandem arrays (see Additional file 1: Tables S11 and S12). Therefore, the asexual genome appears to have a lower abundance of repetitive DNA relative to the sexual genome, in contrast to some predictions for recently evolved asexual genomes.

We next looked for the presence of what had originally been annotated as “missing genes” in the asexual reference genome. Of the 355 “missing *Trichogramma* genes” that were recovered through species-specific blast searches, 348 were also recovered in the sexual genome, indicating that the pattern of “missing genes” (which are actually rapidly evolving genes) is not a result of the transition to asexuality. Therefore, we conclude that the apparent gene loss in *Trichogramma pretiosum* was not due to unusual gene losses or divergence in the asexual lineage, but reflects primarily rapid protein evolution in the *Trichogramma pretiosum* clade.

**Fig. 4:**
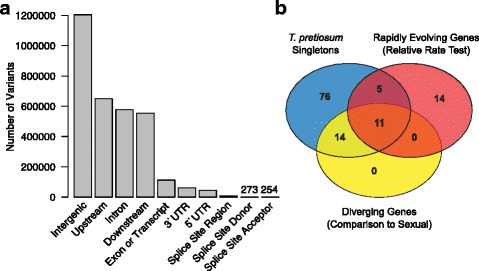
Re-sequencing of a sexual *Trichogramma pretiosum*. **a** Location of variants identified in the sexual *Trichogramma pretiosum* genome with respect to the asexual reference. **b** GO terms identified as diverging in the re-sequencing analyses, and the overlap with GO terms overrepresented in the singleton and rapid protein evolution categories

To better understand the differences between the sexual and asexual *Trichogramma* genomes, we mapped the sexual CA-29 draft genome to the asexual reference. One-to-one mappings spanned 171,208,387 bp of the asexual genome, across 171,190,506 bp of the sexual genome. We identified 1,350,662 single nucleotide polymorphisms (SNPs) and 621,340 indels between the two assemblies for which we determined potential functional consequences. We found 3.5% of the SNPs and indels inside of coding regions (exons and transcripts), and another 3.4% were located within untranslated regions (UTRs) of annotated genes (Fig. 4a). Within protein-coding regions, the missense/silent ratio was 0.5773, indicating a faster rate of evolution, consistent with the longer branch leading to *Trichogramma pretiosum* in the phylogenetic reconstruction, and the high numbers of rapidly evolving genes. However, these genome-wide differences at the nucleotide level cannot reliably be assigned to the asexual versus sexual line without a closely related outgroup. The SNPs and indels constitute just over 1% of all the sites in the reference genome.

We looked for overrepresented functional categories within groups of genes containing different types of variants between the sexual and asexual lines. The most highly diverged genes, as measured by genes in the top 5% of missense mutations per amino acid site, were overrepresented for GO terms also overrepresented in the asexual *Trichogramma pretiosum* singleton gene set (see Additional file 7: Table S18), many of which we went on to show are not actually lineage-specific. More than half of the GO terms overrepresented in the diverged gene category were related to nucleotide metabolism or nitrogen metabolism, and may reflect the egg-feeding ecology of the wasps or the metabolically expensive large brain size in *Trichogramma*. We hypothesized that these rapidly diverging genes were more likely to be categorized as singletons in the comparative analyses. Indeed, a significant portion (51.8%) of the genes undergoing rapid divergence between the sexual and asexual lineages were defined as singletons (*p* < 0.0001), which again is in agreement with the idea that *Trichogramma* singletons may have a longer evolutionary history of divergence that prevented orthology detection in our analyses. We show that the functional categories diverging between the two *Trichogramma pretiosum* lines are also represented in the singleton gene categories, rapidly evolving gene categories (as determined by Tajima’s relative rate test and comparisons to other Hymenoptera), or both (Fig. 4b). Genes with frameshift mutations between the sexual and asexual lines were overrepresented for seven GO terms, five of which were also overrepresented in the divergent category (see Additional file 7: Table S18).

We then quantified dS and dN between the sexual and asexual lines. These measurements also cannot be assigned to the respective lineage without a more closely related outgroup (due to nucleotide substitution saturation). For the single-copy core genes previously used for Tajima’s relative rate testing (*n* = 3180), dS between the sexual and asexual genomes was 0.013 +/− 0.0003. When all *Trichogramma* coding sequences were considered, dS was 0.014 +/− 0.0002. Genes with dN/dS greater than 1 (*n* = 357) were overrepresented for a suite of GO terms (see Additional file 7: Table S18) that again overlap with the GO terms overrepresented in other analyses of rapid evolution and divergence: the regulation of transcriptional processes and nucleotide metabolism. The estimates of dS between the sexual and asexual lines (~ 0.014) are similar to estimates of dS between some sibling species of *Nasonia* [38], and thus represent a high level of divergence for an intraspecific comparison. While it is possible that there are cryptic species within the *Trichogramma pretiosum* clade, it is notable that these two lines readily produce heterozygous (F1) and recombinant (F2) offspring without any apparent hybrid breakdown [57], despite their divergence and geographically distant origins (California and Peru). There is appreciable interspecific hybrid incompatibility within the *Nasonia* clade, and the genes with elevated dN/dS in those comparisons are overrepresented for mitochondrial-interacting genes and are involved in hybrid breakdown [75, 76]. In contrast, mitochondrial gene ontologies are not overrepresented in the rapidly evolving *Trichogramma* gene sets, but were identified as slower evolving in *Trichogramma* compared to the other chalcids (Fig. 3d)*,* which may explain the hybrid compatibility despite the higher levels of nuclear genome divergence.

We next compared rates of protein evolution with the core set of conserved hymenopteran orthologs, using *Nasonia* as the outgroup. While we did not have a closely related outgroup that allowed us to compare evolution of the sexual and asexual genomes at the nucleotide level, using Tajima’s relative rate test allowed us to determine whether or not the divergence between sexual and asexual lineages is a result of more amino acid changes on one branch than the other. There were no proteins with significantly elevated rates of evolution in one branch over the other, indicating that both the sexual and asexual lines are diverging from the common ancestor at similar rates. Thus, divergence between the sexual and asexual lines, and from the rest of Hymenoptera, cannot be attributed to their different reproductive modes but is a feature of the *Trichogramma* clade more generally.

### Methylation in the *Trichogramma pretiosum* genome

**Fig. 5:**
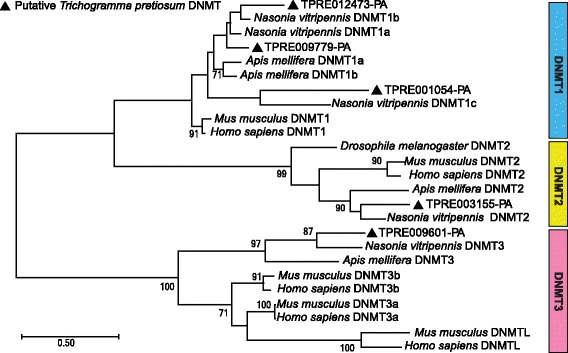
Putative DNA methyltransferases in *Trichogramma pretiosum*. Bootstrap values > 70 are shown

**Fig. 6:**
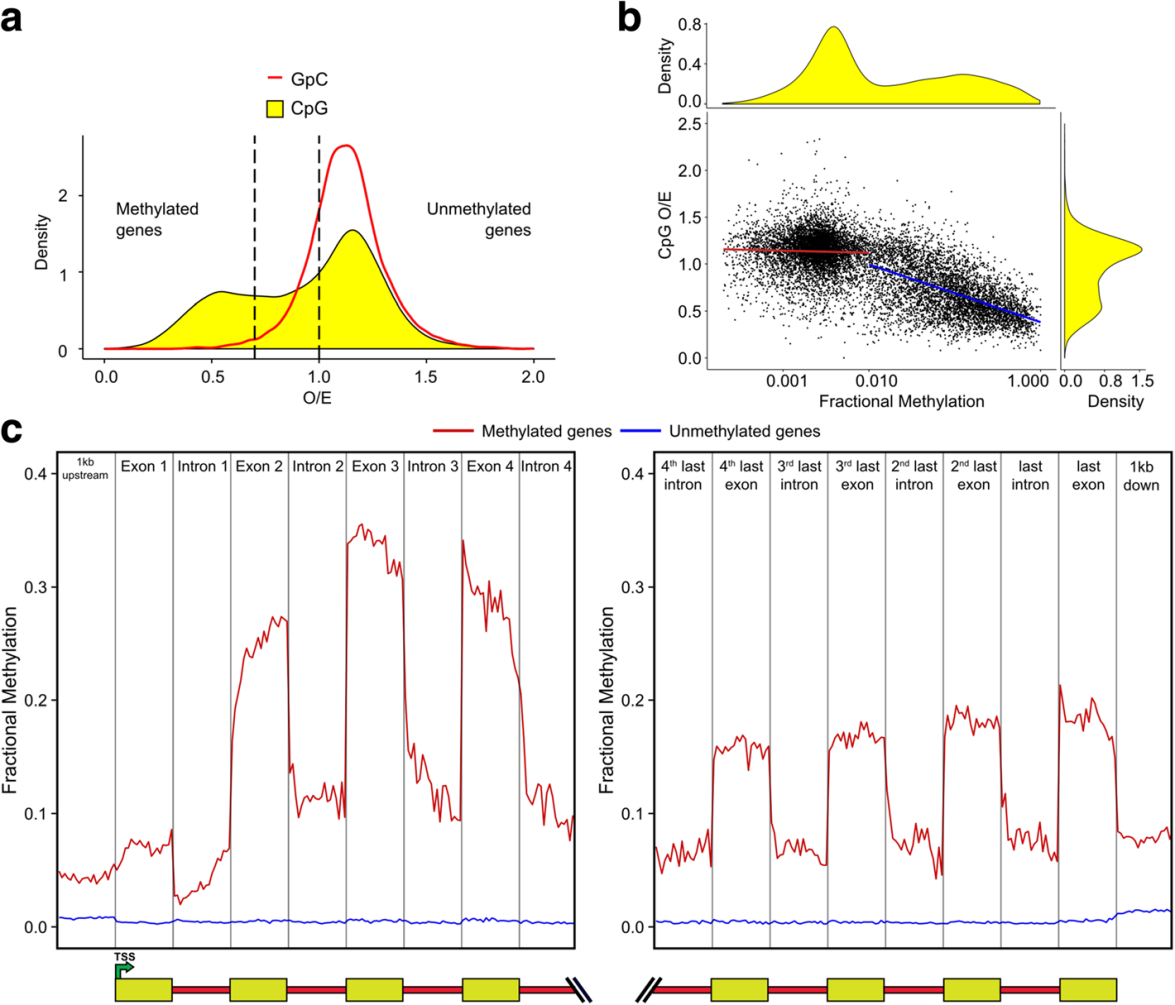
Evidence for methylation in *Trichogramma pretiosum*. **a** Coding sequence (CDS) CpG and GpC observed/expected (*O/E*) densities. **b** Fractional CpG methylation of *Trichogramma pretiosum* genes (log transformed) plotted against gene CDS CpG O/E. *Blue regression line* fit for genes with > 0.01 fractional methylation, *r* = − 0.49; *red regression line* fit for genes with < 0.01 fractional methylation, *r* = 0.051. **c** Average fractional methylation in the first and last four exons and introns of methylated genes and unmethylated genes

DNA methylation is common across many insects, including hymenopterans [77]. In *Trichogramma*, there is evidence that epigenetic patterning is important for sex determination and potentially *Wolbachia*-mediated parthenogenesis [33, 34, 36]. Here, we explore methylation in the reference asexual *Trichogramma pretiosum* genome. We find in total five putative DNA methyltransferases (DNMTs) in the *Trichogramma pretiosum* genome: three DNMT1 orthologs, one DNMT2 ortholog, and one DNMT3 ortholog (Fig. 5). The number of DNMT orthologs is the same as that found in *Nasonia vitripennis* [38]. We used genome-wide distribution patterns of the dinucleotide CpG to detect signatures of methylation. Methylated cytosines (methylcytosines) are mutagenic and often occur in the CpG context, so finding fewer than expected CpG sites is indicative of regions in which methylation occurs [78]. The distinctive bimodal CpG depletion pattern suggests division of methylated and unmethylated genes within the *Trichogramma pretiosum* genome (Fig. 6a). In contrast, the GpC observed/expected (O/E) control (without mutagenic methylcytosines) does not show such a division. It is of interest that the mean GpC O/E is higher than 1 in this genome. In the honey bee genome, the mean CpG O/E is significantly greater than 1 [79]. Subsequent studies analyzing forces underlying nucleotide composition in the honey bee genome have hypothesized that recombination and biased gene conversion might be able to partially explain the excess of CpGs [80, 81]. To investigate the excess of CpGs and GpCs further, we looked at CpG and GpC O/E across seven hymenopterans with well-assembled genomes and show that at least five of these species have the same characteristic of mean GpC O/E > 1, including *Trichogramma pretiosum* (see Additional file 1: Section S3.2, Figure S3). In the absence of well-characterized recombination data, it is difficult to say with any certainty that this is caused by recombination. However, the fact that we observe this in several other species indicates that it is a potentially widespread phenomenon, at least in Hymenoptera.

We experimentally validated methylation patterns in the genome with whole genome bisulfite sequencing and found that gene methylation density mirrors gene CpG O/E density (Fig. 6b). There is a strong negative correlation between gene coding DNA sequence (CDS) CpG O/E and gene fractional methylation, especially for genes above a threshold of 0.01 fractional methylation. For methylated genes (> 0.01 gene body fractional methylation), we confirmed methylation bias towards the 5′ prime end and enrichment of CpG methylation in exons compared to introns (Fig. 6c). This pattern is absent in unmethylated genes and mirrors similar patterns observed in other hymenopterans such as *Nasonia vitripennis* and *Apis mellifera* [82, 83].

### Conservation of methylation

**Fig. 7:**
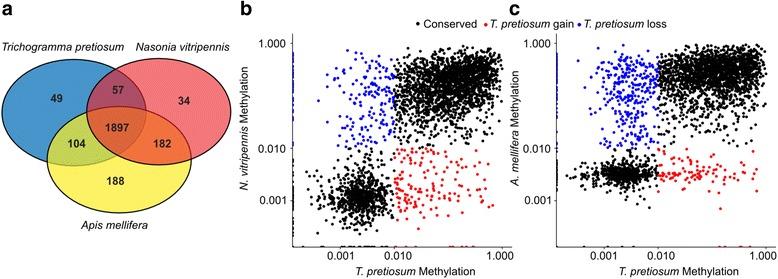
Conservation of methylation across hymenopteran species. **a** Venn diagram depicting methylated orthologs between *T. pretiosum*, *N. vitripennis*, and *A. mellifera*. **b**, **c** *T. pretiosum* lineage-specific methylation gain and loss compared to (**b**) *N. vitripennis* and (**c**) *A. mellifera* orthologs

In *Trichogramma pretiosum*, 4853 genes in the genome are methylated (37.4%). Further, 2107 of the 3180 (66.3%) three-lineage core genes are methylated, meaning that methylated genes are more likely to be phylogenetically conserved (chi-squared statistic: *p* < 0.0001), which is in agreement with previous studies [84, 85]. The majority (*n* = 1897) of the 3180 three lineage core orthologs remain methylated across *Trichogramma pretiosum*, *Nasonia vitripennis*, and *Apis mellifera* (Fig. 7a), and 2566 (80.7%) retain their methylation status (either all methylated or all not methylated). Table 6 summarizes the methylation status changes of *Trichogramma pretiosum* orthologs. We found no significantly overrepresented GO terms for *Trichogramma pretiosum* lineage-specific genes that gained or lost gene body methylation following multiple testing correction. Conserved methylated genes across all three lineages were enriched for GO terms relating to basic cellular functions such as RNA and protein metabolic processes, translation, and protein transport. This is consistent with the general observation that DNA methylation in insects is associated with constitutive gene expression across tissues and developmental stages [82, 83]. Additionally, gene body methylation is positively correlated between orthologs. Log-transformed fractional methylation values compared between *Trichogramma pretiosum* and *Nasonia vitripennis* or *Apis mellifera* are shown in Fig. 7b, c. Correlations were significant in both cases (*Trichogramma pretiosum* - *Nasonia vitripennis*: *r* = 0.50, *p <* 0.001; *Trichogramma pretiosum* - *Apis mellifera*: *r* = 0.47, *p <* 0.001).

**Table 6:** Methylation status of gene body methylation across 3180 single-copy orthologs

Methylation status			Number of orthologs^a^	Interpretation
*T. pretiosum*	*N. vitripennis*	*A. mellifera*		
Methylated	Methylated	Methylated	1897	Conserved methylation status
Methylated	Unmethylated	Unmethylated	49	*T. pretiosum* lineage-specific gain of methylation
Unmethylated	Methylated	Methylated	182	*T. pretiosum* lineage-specific loss of methylation
Unmethylated	Unmethylated	Unmethylated	669	Conserved methylation status

^a^The 383 genes not included in the table are those which have *Apis mellifera*- or *Nasonia vitripennis*-specific gains or losses

### Intron size

**Fig. 8:**
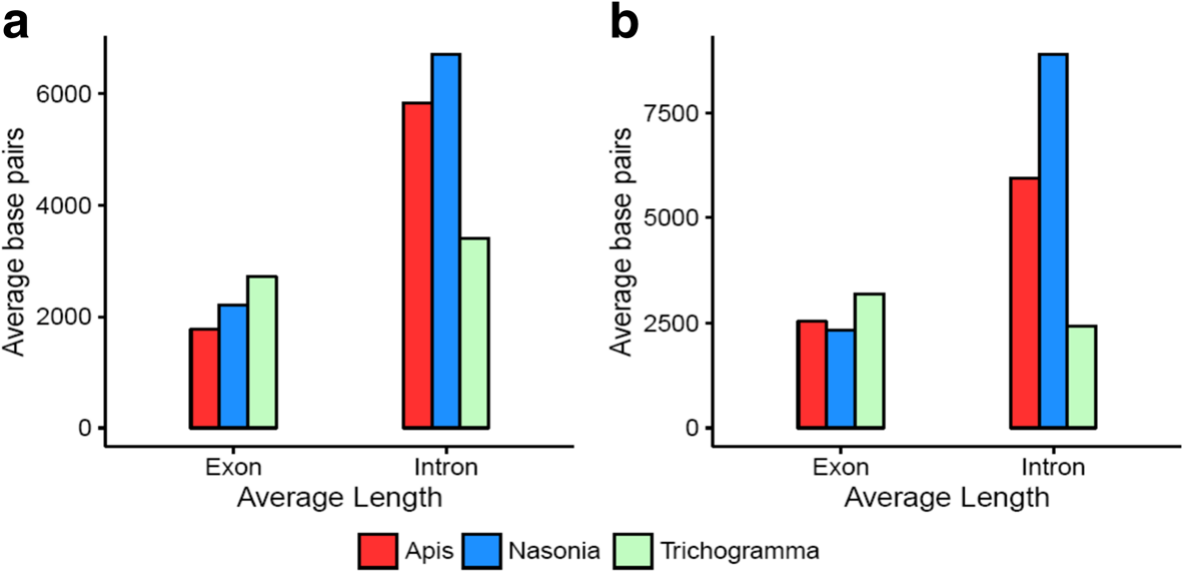
Average *Apis mellifera*, *Nasonia vitripennis*, and *Trichogramma pretiosum* exon and intron lengths. For **a** all genes, **b** 3180 single-copy orthologs

We compared total exon and intron lengths per gene across several hymenopteran species and noted a larger discrepancy in length distributions in *Trichogramma pretiosum*. Mean exon length is longer in *Trichogramma pretiosum*, while mean intron length is noticeably shorter compared to other species (Fig. 8a). To obtain a better understanding of exon/intron size variation, we examined exon and intron sizes of the single-copy core orthologs from *Apis*, *Nasonia*, and *Trichogramma*, used for rate testing and methylation conservation (*n* = 3180) (Fig. 8b). *Trichogramma* is the only lineage where the average intron length is shorter than the average exon length per gene (3405 bp versus 3532 bp, respectively). We detected significant differences in the exon lengths (analysis of variance (ANOVA), *p* < 0.01) and intron lengths (ANOVA, *p* < 0.001) between all lineages. Differences in intron sizes are reflected in the genome size, which is in agreement with previous research [86]. The causes of exon size difference, however, are not yet clear. We speculate that the rapid generation time and very small size of these insects could select for global reductions in intron size, and this finding parallels the discovery that *Trichogramma* genes involved in transcription, gene expression, and RNA metabolism are rapidly evolving. Indeed, intron loss has previously been associated with the evolution of parasitic genomes [87]. We note, however, that although *Trichogramma* has smaller introns, the overall genome size and repetitive fraction in these wasps (~ 200 Mbp, ~ 30%) are not unusually small or low compared to those of many other insects [88], and other *Trichogramma* spp. are estimated to have genome sizes closer to 250 Mbp [9]. Thus, miniaturization has not resulted in an overall reduction in genome size.

## Discussion

*Trichogramma* have been extensively studied as biological control agents, for their relationship with parthenogenesis-inducing *Wolbachia* symbionts, and for their minute size. We obtained a high-quality reference genome for an asexual line of *Trichogramma pretiosum* and found that approximately a third of all the coding sequences have signatures of rapid evolution compared to other hymenopteran species, either through rate testing, protein branch lengths, or the discovery of more divergent orthologs that initially failed to cluster with other hymenopteran proteins. Comparisons at different phylogenetic distances (intraorder and intraspecific) revealed that rapidly evolving *Trichogramma* genes were overrepresented for a number of functional categories, largely nucleic acid metabolism and transcriptional regulation. Genome-wide comparisons to a sexual line of *Trichogramma pretiosum* reveal that the signatures of rapid evolution and apparent gene loss are not due to the transition to asexuality. Genes originally identified as missing were recovered at the same frequency in both the sexual and asexual genomes, indicating no difference due to reproductive mode. Most “missing genes” and “species-specific genes” are neither missing nor species-specific, but are divergent enough that orthology was not detected. Furthermore, we identified no proteins that had a significantly elevated rate of evolution in either the sexual or asexual lineage as compared to *Nasonia*, indicating that they are diverging from their common ancestor at similar rates.

Gene family contractions were more common in chalcids compared to the other hymenopterans, and from this study we detect many chalcid-specific gene family gains and losses that may underlie the rapid adaptive radiation of the chalcid superfamily, or the evolution of a miniaturized ancestor. The most notable of these are the loss of conserved genes involved in signaling, embryonic development, and patterning. In parallel, we identified genes that are more rapidly evolving in chalcids that may also be associated with the evolution of a miniaturized chalcid ancestor. These include functions relating to cell size, cell proliferation, and ploidy.

*Trichogramma pretiosum* has a functional methylation toolkit, and has characteristic patterns of CpG depletion and methylation throughout the genome. The pattern of methylation is phylogenetically conserved across Hymenoptera, with five prime exons consistently being the targets of methylation, and methylation occurring in conserved core genes. Epigenetic patterning likely plays a critical role in sex determination and may be mediated by *Trichogramma*’s relationship with *Wolbachia* [33–35]. Indeed, *Wolbachia* has been shown to affect methylation in other insect hosts [89, 90]. Mechanistic investigation into how *Wolbachia* may sculpt methylation patterns is a promising direction for understanding host-symbiont interactions in this system.

## Conclusions

The *Trichogramma pretiosum* genomes provide the framework for more detailed studies on the basis of symbiont-mediated parthenogenesis, sex determination, and the constraints of parasitism. A deeper understanding of genome adaptations specific to the *Trichogramma* clade versus those associated with sexual-asexual transitions will emerge from additional genome sequencing. The *Trichogramma* system is rich in multiple examples of sexual-asexual transitions resulting in polymorphic and fixed asexual populations of differing divergence for these future studies [5, 19, 30, 91].

## Methods

### Genome sequencing

*Trichogramma pretiosum* is one of 30 arthropod species sequenced as a part of the pilot project for the i5K 5000 arthropod genomes project at the Baylor College of Medicine Human Genome Sequencing Center (HGSC). We sequenced the genome of an irreversibly asexual line of *Trichogramma pretiosum* using an Illumina-ALLPATHS-LG sequencing and assembly strategy. In brief, we generated paired-end 100-bp sequences for libraries of nominal insert sizes 180 bp, 500 bp, 1 kb, 3 kb, and 8 kb, assembled with ALLPATHS-LG (v35218) [92] and further scaffolded and gap-filled using in-house tools Atlas-Link (v.1.0) and Atlas gap-fill (v.2.2) (https://www.hgsc.bcm.edu/software/). The genome was annotated with a Maker 2.0 annotation pipeline tuned specifically for arthropods [93], using RNA-seq libraries to identify exon-intron boundaries. For specific methodological details about biological samples, libraries, sequencing, and annotation, see Additional file 1: Section S1 [5, 29, 30, 58, 59, 92–99].

### Comparative genomics

We reconstructed a phylogeny of 21 hymenopteran species from transcriptomic or genomic data, using 107 single-copy protein-coding genes with RAxML version 8.2.8 [100], and rooted on *Athalia rosae*. This phylogeny was pruned to eight species for which we performed in-depth genomic comparisons (Table 1). It was essential to construct a more complete phylogeny of Hymenoptera, followed by pruning, in order to avoid long branch length attraction, which otherwise resulted in phylogenetic relationships not supported by other phylogenetic studies of Hymenoptera [2, 3, 46, 47, 101]. Protein-coding sequences from these genomes were clustered into families of orthologous genes using OrthoMCL [48]. GO terms were mapped to the coding sequences from all genomes using Blast2GO [102]. To obtain GO terms for each gene family delineated by OrthoMCL, all GO terms represented by at least 40% of the members within a gene family were recorded, as was done in the work of Grbic et al. [103]. We used CAFE v3.1 [104] to identify significantly expanding and contracting gene families across Hymenoptera. For more specific comparative and statistical methods, see Additional file 1: Sections S2.1–S2.4 [38–40, 45–48, 79, 102–113].

### Protein rate evolution: Tajima’s relative rate tests

Protein rate evolution tests were performed with Tajima’s relative rate test [114], using the R package pegas [115], on amino acid alignments of single-copy orthologs, comparing *Trichogramma pretiosum* to *Nasonia vitripennis*, using *Apis mellifera* as an outgroup. Alignments were created with MAFFT v7.271 [106], and rate testing was performed on two versions of the alignments: those masked of especially divergent regions with Gblocks [107, 116], and full-length alignments of the same orthologs. BiNGO [111] was used to identify significantly overrepresented GO terms in all analyses. For more specific comparative and statistical methods, see Additional file 1: Section S2.5 [106, 107, 111, 114–116].

### Protein rate evolution: phylogenetic comparisons

In addition to the pairwise comparisons between *Trichogramma* and *Nasonia* proteins (*n* = 3180), we compared rates of protein evolution across all species in our hymenopteran phylogeny for each protein in the single-copy core hymenopteran genome (*n* = 1311 proteins). Based on the previously constructed species tree, branch lengths of the alignments masked by Gblocks were estimated with RAxML version 8.2.11 [100], using the same parameters as those used for construction of the species tree. Branch lengths were extracted using Newick Utilities version 1.6 [117]. Raw distances from each chalcid species to the chalcid root and from each hymenopteran species to the hymenopteran root were ordered in R v3.4.1 [118], so as to identify the protein with the longest branch length within each orthologous group. For each chalcid, we normalized branch lengths by dividing the “species-to-chalcid root distance” by the distance from the chalcid root to the hymenopteran root. This normalization better allowed for comparisons between proteins, accounting for the background rate of evolution (as determined by the distance from the chalcid root to the hymenopteran root). Additionally, we ranked all 1311 proteins by their raw branch lengths—within each chalcid genome using the “species-to-chalcid root distance” and separately by the “chalcid root-to-hymenopteran root” distance. GO term analyses were performed as described in Additional file 1: Section S2. Where indicated in the results, chi-squared analyses were performed with the fifer package in R v3.3.2, using the chisq.post.hoc function [119].

### Methylation in *Trichogramma pretiosum*

We collected previously described DNMT protein sequences and identified putative DNMTs in *Trichogramma pretiosum.* Clustal Omega [120] was used to align the sequences, and Molecular Evoutionary Genetics Analysis (MEGA) [121] was used to construct a maximum likelihood tree. Computational predictions of methylation in the *Trichogramma pretiosum* genome were performed using the nucleotide composition method (CpG O/E method): CpG observed/expected and GpC observed/expected were calculated for CDS [122]. Methylation patterns were experimentally validated via whole genome bisulfite sequencing. See Additional file 1: Section S3 [38, 120–125] for additional details on methylation analyses and sequencing.

### Intron size

We compared total exon and intron lengths per gene across *Trichogramma pretiosum*, *Apis mellifera*, and *Nasonia vitripennis.* Additionally, we compared intron and exon lengths for the genes that are single copy, as determined by our OrthoMCL analyses, in the three genomes. One-way ANOVA followed by Tukey’s honest significant difference (HSD) test was used to determine significant differences between lineages for intron and exon lengths.

### Comparisons to sexual *Trichogramma pretiosum*

*Trichogramma pretiosum* contains both sexual and asexual forms; populations can either be a mixture of types or fixed for either sexual or asexual forms [5, 19, 30, 91]. For an initial comparison of sexual and asexual strains, we performed whole genome shotgun sequencing for a sexual line of *Trichogramma pretiosum*, CA-29, a previously described inbred line [57]. In contrast to the reference asexual line from Peru, CA-29 originates from Irvine, California, where populations of *Trichogramma pretiosum* are completely sexual and have not been collected with *Wolbachia* infections [19]. Critically, these two lines (Insectary and CA-29) are compatible and have no obvious reductions in fitness in either the F1 or F2 generation [57]. We generated 27,480,751 paired-end, 250-bp reads, amounting to ~ 70× coverage of a 195-Mbp genome. We performed an assembly with MaSuRCA [60], aligned the CA-29 draft genome to the reference asexual i5k genome and called differences with NUCmer [62], and identified functional consequences of SNPs and indels with SnpEff [63]. Additional details are provided in Additional file 1: Section S4 [19, 57–68].

## Additional files

Additional file 1: Supplemental methods and data, **Tables S1**–**S12.**, and **Figures S1**–**S5**. (DOCX 1157 kb)

Additional file 2: **Table S13.** GO terms overrepresented in chalcid-missing and chalcid-unique gene families. (XLSX 87 kb)

Additional file 3: **Table S14.** *Trichogramma* singleton genes identified by OrthoMCL, and the GO terms overrepresented in that set. (XLSX 97 kb)

Additional file 4: **Table S15.** *Trichogramma* proteins identified to be rapidly evolving as compared to *Nasonia*, using Tajima’s relative rate test, along with overrepresented GO terms. (XLSX 119 kb)

Additional file 5: **Table S16.** Branch lengths of core hymenopteran proteins and overrepresented GO terms for sets of genes with the longest and shortest branches in different species. (XLSX 324 kb)

Additional file 6: **Table S17.** Proteins and overrepresented GO terms for within-genome rank comparisons. (XLSX 326 kb)

Additional file 7: **Table S18.** Comparisons to a sexual genome. Proteins and overrepresented GO terms for proteins most diverged between sexual and asexual lines of *Trichogramma*, frameshifted, and dN/dS greater than 1. (XLSX 69 kb)
