# Supplementary material for: Comparative genomics of the miniature wasp and pest control agent Trichogramma pretiosum
Source: BMC Biol. 2018 May 18;16:54. doi: 10.1186/s12915-018-0520-9 (PMC5960102; doi:10.1186/s12915-018-0520-9)
Supplement: Supplementary file 1 — Supplemental methods and data, Tables S1–S12., and Figures S1–S5. (DOCX 1157 kb) [file 12915_2018_520_MOESM1_ESM.docx]

**SUPPLEMENTAL INFORMATION FOR:**

**Comparative genomics of the miniature wasp and pest control agent *Trichogramma pretiosum***

Amelia R.I. Lindsey^1,2*^, Yogeshwar D. Kelkar^3^ , Xin Wu^4^, Dan Sun^4^, Ellen O. Martinson^3,5^, Zhichao Yan^3,6^, Paul F. Rugman-Jones^1^, Daniel S.T. Hughes^7^, Shwetha C. Murali^7^, Jiaxin Qu^7^, Shannon Dugan^7^, Sandra L. Lee^7^, Hsu Chao^7^, Huyen Dinh^7^, Yi Han^7^, HarshaVardhan Doddapaneni^7^, Kim C. Worley^7^, Donna M. Muzny^7^, Gongyin Ye^6^, Richard A. Gibbs^7^, Stephen Richards^7^, Soojin V. Yi^4^, Richard Stouthamer^1*†^, John H. Werren^3*†^

^*^To whom correspondence should be addressed

^†^Denotes equal contribution

^1^Department of Entomology, University of California Riverside, Riverside, California, 92521

^2^Current Address: Department of Biology, Indiana University, Bloomington, Indiana, 47405

^3^Biology Department, University of Rochester, Rochester, New York, 14627

^4^School of Biological Sciences, Institute for Bioengineering and Bioscience, Georgia Institute of Technology, Atlanta, Georgia 30332

^5^Current address: Department of Entomology, University of Georgia, Athens, Georgia 30602

^6^State Key Laboratory of Rice Biology & Ministry of Agriculture Key Laboratory of Agricultural Entomology, Institute of Insect Sciences, Zhejiang University, Hangzhou, China, 310058

^7^Human Genome Sequencing Center, Department of Molecular and Human Genetics, Baylor College of Medicine, Houston, Texas, 77030

**TABLE OF CONTENTS**

**S1. Genome Sequencing, Assembly, & Annotation**

**SI.1** **DNA and RNA for Sequencing and Annotation**

**S1.2 Genome Sequencing**

**S1.3 Gene Annotation**

**S2. Comparative Genomics**

**S2.1 Hymenopteran Phylogenetics**

**S2.2 Other Genomes and Gene Family Clustering**

**S2.3 Gene Ontology Assignments**

**S2.4 Gene Family Expansions and Contractions**

**S2.5 Protein Evolution**

**S2.6 Lineage Specific Gene Searches**

**S3. Methylation**

**S3.1 DNMT Identification**

**S3.2 Computational Predictions of Methylation**

**S3.3 Bisulfite Sequencing and Analysis**

**S3.4 Conservation of Methylation**

**S4. Comparisons to a Sexual *Trichogramma pretiosum***

**S4.1** **Biological Materials, Sequencing, and Assembly**

**S4.2 Alignment and Comparison to the Reference**

**S4.3 Divergence (dN/dS) Estimates**

**S5. Immunity**

**S5.1 Immune Gene Searches**

**S5.2 Immunity Results**

**S6. Repetitive Elements**

**S6.1 K-mer Analysis to Estimate Repeat Compositions**

**S6.2 K-mer Based Inference of Transposable Elements**

**S1. GENOME SEQUENCING, ASSEMBLY, & ANNOTATION**

**S1.1** **DNA and RNA for Sequencing and Annotation**

An asexual line of *Trichogramma* *pretiosum* was mass reared for sequencing. This colony has been maintained in a commercial insectary since 1966 (Beneficial Insectary, Guelph, Ontario, Canada), after collection from the Puira Valley of Peru. Laboratory replicates of this colony are maintained in 12 x 75 mm glass culture tubes stopped with cotton and incubated at 24^o^C, L:D = 16:8, with 50% relative humidity. Every 10 days cultures are offered fresh honey and egg cards made of irradiated *Ephestia kuehniella* host eggs (Beneficial Insectary, Guelph, Ontario, Canada) adhered to card stock with double-sided tape. Species identification was confirmed by molecular protocols from [Stouthamer, et al. [](#_ENREF_1)65], and *Wolbachia* infection status was confirmed through antibiotic curing [[5](#_ENREF_2)] and PCR [[66](#_ENREF_3)]. Establishment of a *Wolbachia*-free replicate of this colony has not been possible, as females do not fertilize their eggs at high enough frequencies, rendering them dependent upon *Wolbachia,* as seen in [Russell and Stouthamer [](#_ENREF_4)30].

DNA was extracted from ~2,000 males obtained by treating a single generation of the Insectary line with antibiotics using procedures from [[5](#_ENREF_2)]. The single dose of antibiotics is strong enough to knock down *Wolbachia* titers to a level that results in the production of predominantly male offspring in the following generation, but does not eliminate *Wolbachia* completely. This was done to prevent sequencing libraries from being saturated with *Wolbachia* reads, as *Wolbachia* titers can be extremely high in *Trichogramma* [29, 101-102]. Approximately 2,000 infected males (10 mg) were collected and euthanized in 100% ethanol in a glass Dounce tissue grinder tube. The ethanol was subsequently decanted and the sample allowed to air dry before being homogenized in 180 μl of PBS using a glass pestle. The homogenate was transferred to a 1.5 ml microcentrifuge tube and DNA was extracted using the QIAGEN® DNeasy Blood and Tissue Kit following the manufacturer’s protocol for purification of total DNA from insects (available at: [www.qiagen.com/literature/render.aspx?id=528](http://www.qiagen.com/literature/render.aspx?id=528)), completed with a final elution of the DNA in 100 μl of Buffer AE. The extraction was replicated 8 times and subsequently combined into a single eluate. Extracted DNA was quantified using a Nanodrop 1000 and diluted to 50 ng/μl. For RNA extractions, additional male wasps were collected, as well as female wasps from a replicate colony of the Insectary line that had not been treated with antibiotics. The male pool and the female pool were separately flash frozen in liquid nitrogen, and RNA was extracted using the QIAGEN® RNeasy Mini Kit, following the manufacturer’s protocol.

**Table S1.** DNA and RNA used for RNAseq and DNA sequencing

| **Sample Type** | **Life Stage extracted** | **Sex** | **Conc. (ng/ul)** | **Vol. (ul)** | **Total (ug)** | **Notes** |
| --- | --- | --- | --- | --- | --- | --- |
| DNA | Adult | Males | 50 | 1100 | 55 | Pool of ~2,000 males obtained through treating mothers with antibiotics |
| Total RNA | Adult | Female | 1608 | 54 | 87 | Pool of ~2,000 females |
| Total RNA | Adult | Male | 331 | 27 | 9 | Pool of ~2,000 males obtained through treating mothers with antibiotics |

**S1.2 Genome Sequencing**

*Trichogramma* *pretiosum* is one of 30 arthropod species sequenced as a part of the pilot project for the i5K 5000 arthropod genomes project at the Baylor College of Medicine Human Genome Sequencing Center. For all of these species, an enhanced Illumina-ALLPATHS-LG sequencing and assembly strategy enabled multiple species to be approached in parallel at reduced costs. For *Trichogramma* *pretiosum* we sequenced libraries of nominal insert sizes 180bp, 500bp, 1kb, 3kb and 8 kb at genome coverages of 108.2X, 71.8X, 27.0X, 85.1X and 55.8X respectively (assuming a 200 Mb genome size). These raw sequences have been deposited in the NCBI SRA, accessions are shown in Table S2, BioSample ID: SAMN02439301.

To prepare the 180bp and 500bp libraries, we used a gel-cut paired end library protocol. Briefly, 1 µg of the DNA was sheared using a Covaris S-2 system (Covaris, Inc. Woburn, MA) using the 180-bp or 500-bp program. Sheared DNA fragments were purified with Agencourt AMPure XP beads, end-repaired, dA-tailed, and ligated to Illumina universal adapters. After adapter ligation, DNA fragments were further size selected by agarose gel and PCR amplified for 6 to 8 cycles using Illumina P1 and Index primer pair and Phusion® High-Fidelity PCR Master Mix (New England Biolabs). The final library was purified using Agencourt AMPure XP beads and quality assessed by Agilent Bioanalyzer 2100 (DNA 7500 kit) determining library quantity and fragment size distribution before sequencing.

Long mate pair libraries with 1kb, 3kb, and 8kb insert sizes were constructed according to the manufacturer’s protocol (Mate Pair Library v2 Sample Preparation Guide art # 15001464 Rev. A PILOT RELEASE). Briefly, 5 µg (for 1kb and 3kb gap size library) or 10 µg (8kb gap size library) of genomic DNA was sheared to desired size fragments by Hydroshear (Digilab, Marlborough, MA), then end repaired and biotinylated. Fragment sizes between 0.8 - 1.5 kb (1kb) 3 - 3.7 kb (3kb) or 8-10 kb (8 kb) were purified from 1% low melting agarose gel and then circularized by blunt-end ligation. These size selected circular DNA fragments were then sheared to 400-bp (Covaris S-2), purified using Dynabeads M-280 Streptavidin Magnetic Beads, end-repaired, dA-tailed, and ligated to Illumina PE sequencing adapters. DNA fragments with adapter molecules on both ends were amplified for 12 to 15 cycles with Illumina P1 and Index primers. Amplified DNA fragments were purified with Agencourt AMPure XP beads. Quantification and size distribution of the final library was determined before sequencing as described above.

Sequencing was performed on Illumina HiSeq2000s generating 100bp paired end reads. Reads were assembled using ALLPATHS-LG (v35218) [[99](#_ENREF_8)] and further scaffolded and gap-filled using in-house tools Atlas-Link (v.1.0) and Atlas gap-fill (v.2.2)(https://www.hgsc.bcm.edu/software/). This yielded an assembly of size 195Mb with contig N50 of 78.6kb and scaffold N50 of 3.7Mb. The assembly has been deposited in the NCBI: BioProject PRJNA168121. Read coverage (using the 180bp-insert library) confirmed that the average scaffold coverage was ~100X (Figures S1 and S2).

**Figure S1.** Histogram of average read coverage per assembled scaffold. The small number of especially high coverage scaffolds is expected and due to pile up of repetitive elements.

**Figure S2.** Removal of the upper outlier scaffolds better shows the distribution of read coverage. The lowest coverage scaffold is the *w*Tpre *Wolbachia* genome, which was previously published and relocated to a separate GenBank record [[40](#_ENREF_9)].

**S1.3 Gene Annotation**

*Trichogramma pretiosum* is one of 30 i5K pilot genome assemblies that were subjected to automatic gene annotation using a Maker 2.0 annotation pipeline tuned specifically for arthropods. The pipeline is designed to be systematic providing a single consistent procedure for the species in the pilot study, scalable to handle 100’s of genome assemblies, evidence guided using both protein and RNA-seq evidence to guide gene models, and targeted to utilize extant information on arthropod gene sets. The core of the pipeline was a Maker 2 instance, modified slightly to enable efficient running on our computational resources [100]. The genome assembly was first subjected to de-novo repeat prediction and CEGMA [103] analysis to generate gene models for initial training of the ab-initio gene predictors. Three rounds of training of the Augustus [104] and SNAP [105] gene predictors within Maker were used to bootstrap to a high quality training set. Input protein data included 1 million peptides from a non-redundant reduction (90% identity) of Uniprot Ecdysozoa (1.25 million peptides) supplemented with proteomes from eighteen additional species (*Strigamia maritima, Tetranychus urticae, Caenorhabditis elegans, Loa loa, Trichoplax adhaerens, Amphimedon queenslandica, Strongylocentrotus purpuratus, Nematostella vectensis, Branchiostoma ﬂoridae, Ciona intestinalis, Ciona savignyi, Homo sapiens, Mus musculus, Capitella teleta, Helobdella robusta, Crassostrea gigas, Lottia gigantea,* and *Schistosoma mansoni*) leading to a final nr peptide evidence set of 1.03 million peptides. RNA-seq from *Trichogramma pretiosum* adult males and females was used judiciously to identify exon-intron boundaries but with a heuristic script to identify and split erroneously joined gene models. We used CEGMA models for QC purposes: for *Trichogramma pretiosum*, of 1,977 CEGMA single copy ortholog gene models, 1,929 were found in the assembly, and 1,887 in the final predicted gene set. Additionally, genome completeness was assessed with BUSCO [106] using coding sequences (-m OGS), and comparisons to the arthropod dataset. Finally, the pipeline uses a nine-way homology prediction with human, *Drosophila* and *C. elegans*, and InterPro Scan5 to allocate gene names. The automated gene set is available from the BCM-HGSC website (<https://www.hgsc.bcm.edu/arthropods/parasitic-wasp-t-pretiosum-genome-project)> and at the National Agricultural Library (<https://i5k.nal.usda.gov>)

**Table S2:** Sequencing, assembly, annotation statistics and accession numbers

| Bio Projects | i5K Pilot NCBI Bio-project | PRJNA163973  http://www.ncbi.nlm.nih.gov/bioproject/163973 |
| --- | --- | --- |
|  | *Trichogramma pretiosum* NCBI Bio-project | PRJNA168121  https://www.ncbi.nlm.nih.gov/bioproject/168121 |
|  | NCBI Bio-sample | SAMN02439301  https://www.ncbi.nlm.nih.gov/biosample/2439301 |
| Genome Sequence | 180bp insert (insert size 203bp, sd 25 bp) | 1 run, 102.2M spots, 20.6 Gbp |
|  | 500bp insert (insert size 420bp sd 41 bp) | 1 run, 71.1M spots, 14.4 Gbp |
|  | 1kb insert (not available) | 1 run, 26.8M spots, 5.4 Gbp |
|  | 3kb insert (insert 2,989 bp, sd 351 bp) | 1 run, 84.3M spots, 17.0 Gbp |
|  | 8kb insert (insert 7,255 bp, sd 2,251 bp) | 1 run, 55.3M spots, 11.2 Gbp |
|  | 180bp insert NCBI SRA Accession | SRX488821  http://www.ncbi.nlm.nih.gov/sra/SRX488821 |
|  | 500bp insert NCBI SRA Accession | SRX488825  http://www.ncbi.nlm.nih.gov/sra/SRX488825 |
|  | 1000bp insert NCBI SRA Accession | SRX488824  https://www.ncbi.nlm.nih.gov/sra/SRX488824 |
|  | 3kb insert NCBI SRA Accession | SRX488823  http://www.ncbi.nlm.nih.gov/sra/SRX488823 |
|  | 8kb insert NCBI SRA Accession | SRX488822  http://www.ncbi.nlm.nih.gov/sra/SRX488822 |
| Genome Assembly | Number of contigs | 7,879 |
|  | Contig N50 | 78,655 bp |
|  | Number of scaffolds | 357 |
|  | Scaffold N50 | 3,706,225 bp |
|  | Size of final assembly | 195,087,592 bp |
|  | Size of final assembly - without gaps | 180,028,424 bp |
|  | NCBI Genome Assembly Accession | GCA_000599845.2 https://www.ncbi.nlm.nih.gov/assembly/GCA_000599845.2 |
| RNAseq Data | Adult Male RNAseq reads | 25.9M spots, 5.2G bases |
|  | Adult Female RNAseq reads | 21.9M spots, 4.4G bases |
|  | Adult Male RNAseq SRA Accession | SRX897636  http://www.ncbi.nlm.nih.gov/sra/SRX897636 |
|  | Adult Female RNAseq SRA Accession | SRX897635  http://www.ncbi.nlm.nih.gov/sra/SRX897635 |
| Bisulfite Seq Data | Adult Femae BisSeq reads | 111M spots, 16.3G bases |
|  | Adult Female BisSeq SRA Accession | SRX2379827  http://www.ncbi.nlm.nih.gov/sra/SRX2379827 |
| Automated Genome Annotation (TPRE_0.5.3) | Genes (TPRE_0.5.3) | 12,928 (13,200 including *Wolbachia* *w*Tpre genome, Scaffold 109) |
|  | Average Transcript length | 2,721 bp |
|  | Average CDS length | 1,536 bp (512 aa) |
|  | Exons per gene | 5.78 |
|  | Genome Annotation Link | National Agricultural Library  https://i5k.nal.usda.gov/Trichogramma_pretiosum |

**S2. COMPARATIVE GENOMICS**

**S2.1 Hymenopteran Phylogenetics**

A species phylogeny based on 107 protein sequences was reconstructed to determine the evolutionary relationships between 21 hymenopterans. The 107 proteins were selected from the official peptide sets of the nine species with available genomes and *de novo* assembled adult female transcriptomes for the remaining 12 species (Table S3). The protein set from each species was searched against the official peptide set of the *Nasonia vitripennis* genome (OGSv2.0 http://arthropods.eugenes.org/genes2/nasonia/genes/) using BLASTp. A significant e-value cut-off ≤ 1 e^-5^ was applied and only genes that had a single hit across all twenty-one species were included in further analysis. Protein sequences were aligned with MAFFT [112] using default settings. Alignments were trimmed using Gblocks to remove gaps [113]. Multiple alignments of the 107 single-copy protein-coding genes were concatenated prior to phylogenetic reconstruction using RAxML version 8.2.8 with the PROTGAMMAWAG model, and 1,000 bootstrap replicates. The tree was rooted on *Athalia rosae*, sister to the hymenopteran species used in our analyses [46,47]. The phylogeny was visualized with FigTree version 1.4.2 (http://tree.bio.ed.ac.uk/software/figtree/), and annotated in Inkscape (<https://inkscape.org/>).

**S2.2 Other Genomes and Gene Family Clustering**

Seven other hymenopteran genomes and their annotations were used in comparative analyses (Table S4). All unpublished i5k genomes were scanned with the [Wheeler, et al. [](#_ENREF_19)114] pipeline to identify potential bacterial contamination. Raw reads were re-aligned to the reference genome in CLC Genomics Workbench v6.0.2 (CLC bio, Aaarhus, Denmark) and scaffold coverage was calculated to aid in identifying bacterial contamination. Scaffolds and associated annotations identified as “bacterial” and sequenced at least at a 10-fold difference of the average coverage were removed from downstream analyses. In the *Athalia rosae* assembly, we identified 39 scaffolds with strong evidence of bacterial contamination. These scaffolds and associated annotations were removed from the comparative analyses. The *Trichogramma pretiosum* assembly contained a near-complete *Wolbachia* genome, Scaffold 109, which was removed and published separately [[114](#_ENREF_9)]. No other evidence for bacterial contamination was found in the other i5k genomes.

**Table S3:** Genomes and transcriptomes used in phylogenetic reconstruction

| **Species** | **Sequence Source** | **Accession** | **Reference** |
| --- | --- | --- | --- |
| *Athalia rosae* | Genome | GCA_000344095.1 | I5k, unpublished |
| *Orussus abietinus* | Genome | GCA_000612105.1 | I5k, unpublished |
| *Apis mellifera* | Genome | GCA_000002195.1 | [Consortium [](#_ENREF_20)86] |
| *Microplitis demolitor* | Genome | GCA_000572035.2 | [Burke, et al. [](#_ENREF_21)115] |
| *Trichogramma pretiosum* | Genome | GCA_000599845.2 | This study |
| *Copidosoma floridanum* | Genome | GCA_000648655.1 | I5k, unpublished |
| *Tachinaephagus zealandicus* | Transcriptome | N/A | Martinson and Werren, unpublished |
| *Melittobia spp.* | Transcriptome | N/A | Martinson and Werren, unpublished |
| *Ceratosolen solmsi* | Genome | GCA_000503995.1 | [Xiao, et al. [](#_ENREF_22)51] |
| *Spalangia endius* | Transcriptome | N/A | Martinson and Werren, unpublished |
| *Spalangia cameronii* | Transcriptome | N/A | Martinson and Werren, unpublished |
| *Muscidifurax raptor* | Transcriptome | N/A | Martinson and Werren, unpublished |
| *Muscidifurax raptorellus* | Transcriptome | N/A | Martinson and Werren, unpublished |
| *Muscidifurax uniraptor* | Transcriptome | N/A | Martinson and Werren, unpublished |
| *Pteromalus puparum* | Genome | N/A | Ye and Werren, unpublished |
| *Urolepis rufipes* | Transcriptome | N/A | Martinson and Werren, unpublished |
| *Trichomalopsis sarcophagae* | Transcriptome | N/A | [Martinson, et al. [](#_ENREF_23)116] |
| *Nasonia vitripennis* | Genome | GCA_000002325.2 | [Rago, et al. [](#_ENREF_24)39] |
| *Nasonia longicornis* | Transcriptome | N/A | Martinson and Werren, unpublished |
| *Nasonia giraulti* | Transcriptome | N/A | Martinson and Werren, unpublished |
| *Nasonia oneida* | Transcriptome | N/A | Martinson and Werren, unpublished |

Following the removal of “bacterial” scaffolds from the seven other hymenopteran genomes used for comparative analysis, protein-coding annotations associated with the remaining scaffolds were obtained (Table S5). For genes with multiple transcript variants, only the longest isoform was used in downstream clustering. After pooling together these protein-coding genes with those of *T. pretiosum,* we used OrthoMCL [48], to identify clusters of ortholgous and paralogous genes. For the initial blastp step of OrthoMCL we used the E-value cut-off value of 10^-10^. The OrthoMCL parameter ‘percentMatchCutoff’ and MCL’s inflation parameter were set to 70 and 2.5 respectively in order to ensure that only proteins that share true orthology or paralogy relationships were clustered together. A total of 14,168 orthologous groups with more than one gene were obtained, out of which 1,311 groups represented genes present as single copies in all eight genomes used.

**Table S4.** Genomes used in comparative analyses

| **Species** | **Common Name** | **Family** | **Superfamily** | **Assembly** | **Reference** |
| --- | --- | --- | --- | --- | --- |
| *Apis*  *mellifera* | Honey Bee | Apidae | Apoidea | AADG00000000 | [86] |
| *Athalia*  *rosae* | Sawfly | Tenthredinidae | Tenthredinoidea | AOFN00000000 | I5k, Unpublished |
| *Ceratosolen solmsi* | Fig Wasp | Agaonidae | Chalcidoidea | ATAC00000000 | [51] |
| *Copidosoma floridanum* | Polyembryonic Wasp | Encyrtidae | Chalcidoidea | JBOX00000000 | I5k, Unpublished |
| *Microplitis demolitor* | Braconid Parasitoid Wasp | Braconidae | Ichneumonoidea | AZMT00000000 | [115] |
| *Nasonia vitripennis* | Jewel Wasp | Pteromalidae | Chalcidoidea | AAZX00000000 | [38] |
| *Orussus abietinus* | Wood Wasp | Orussidae | Orussoidea | AZGP00000000 | I5k, Unpublished |
| *Trichogramma pretiosum* | *Trichogramma* Wasp | Trichogrammatidae | Chalcidoidea | JARR00000000 | This study |

**Table S5.** Genome annotations used in comparative analyses

| **Species** | **Source** | **Accession/version** | **Reference** |
| --- | --- | --- | --- |
| *Apis mellifera* | NCBI RefSeq | PRJNA10625 | [86] |
| *Athalia rosae* | I5k | version_0.5.3 | Unpublished |
| *Ceratosolen solmsi* | NCBI RefSeq | PRJNA277475 | [51] |
| *Copidosoma floridanum* | NCBI RefSeq | PRJNA171748 | Unpublished |
| *Microplitis demolitor* | NCBI RefSeq | PRJNA251518 | [115] |
| *Nasonia vitripennis* | WaspAtlas | EvidentialGene dataset | [39] |
| *Orussus abietinus* | NCBI RefSeq | PRJNA282746 | Unpublished |
| *Trichogramma pretiosum* | I5k | version_0.5.3 | This Study |

**S2.3 Gene Ontology Assignments**

GO terms were mapped to the coding sequences from all genomes using Blast2GO [109] after blastp searches against the NCBI non-redundant database, and interpro scans with the Blast2GO software. To obtain GO terms for each gene family delineated by OrthoMCL, all GO terms represented by at least 40% of the members within a gene family were recorded, as was done in [Grbic, et al. [](#_ENREF_28)110].

**S2.4 Gene Family Expansions and Contractions**

We used CAFE [111] to identify significantly expanding and contracting gene families across Hymenoptera, at a corrected significance level of p = 0.05. The birth-death rate parameter, lambda, was determined with the -s flag that optimizes the log likelihood of data for all families. To avoid long branch length attraction, the 21-species phylogeny was pruned in R, using the ape package [118] to obtain a phylogeny upon which we mapped gene family evolution. BiNGO [117] was used to identify significantly overrepresented GO terms in sets of expanding and contracting gene families. Testing was performed with hypergeometric tests and Benjamini & Hochberg FDR correction, at a corrected significance level of 0.05, with the complete set of gene families and their associated GO terms (see above) as the background. For species-specific genes, the same statistical methods were used, but with the GO terms for individual genes in the respective genome as the background, instead of GO terms for families.

**S2.5 Protein Evolution**

To test for differences in rates of protein evolution between *Trichogramma pretiosum* and other hymenopterans, we used Tajima’s relative rate test [120] as implemented in the R package pegas [121]. Due to the long branch-lengths and divergence times, we used amino acid data to avoid issues of nucleotide substitution saturation. We compared amino acid sequences from *Trichogramma pretiosum* to *Nasonia vitripennis*, using *Apis mellifera* as an outgroup. Protein sequences from all gene families where these three species were single copy (n = 3180) were aligned with MAFFT v7.271 [112] using standard parameters, and Gblocks v0.91b [113,122] was used to mask poorly aligned and especially divergent regions of the alignments. Relative rate testing was performed in R v3.3.2 using both masked and un-masked alignments for comparison. P-values were collected and bonferroni corrections were performed for multiple testing. BiNGO [117] was used to identify significantly overrepresented GO terms in gene families with significantly elevated rates of evolution. Testing was performed with hypergeometric tests and Benjamini & Hochberg FDR correction, at a corrected significance level of 0.05, with the set of single copy gene families (n = 3180) and their associated GO terms as the background. Only the subset that was used for rate testing was used as the background so as to not bias results towards GO terms associated with single-copy gene families.

**S2.6 Lineage Specific Gene Searches**

To identify orthologs of the 4,203 genes originally identified in *Trichogramma pretiosum* to be lineage specific, we used blastp to search for the protein sequence of these genes against a custom database of the seven other hymenopteran species. The top hit for each of the “lineage specific” genes was recorded, and the numbers of genes recovered at various thresholds are reported in Figure 2D of the main manuscript.

**S3. METHYLATION**

**S3.1 DNMT Identification**

We collected DNA methyltransferase (DNMT) protein sequences from *Homo sapiens*, *Mus musculus*, *Apis mellifera*, *Drosophila melanogaster,* and *Nasonia vitripennis*, and used BLAST (2.2.18) to perform the search for *Trichogramma* *pretiosum* DNMTs against the database of these proteins. E-value cutoff was set to 1e-20. Clustal Omega [126] was used to align the sequences and MEGA [127] used to construct a maximum likelihood tree.

**S3.2 Computational Predictions of Methylation**

To look for signatures of methylation in the *Trichogramma* *pretiosum* genome, we used the Nucleotide Composition Method (CpG O/E method): CpG observed/expected and GpC observed/expected were calculated for CDS sequences as previously described [128]. We also looked at CpG and GpC O/E in six other well-assembled hymenopteran genomes (Figure S3).

**Figure S3.** Coding sequence (CDS) CpG and GpC observed/expected (O/E) densities across seven Hymenopteran genomes shows that mean GpC O/E > 1 is not unusual in Hymenoptera.

**S3.3 Bisulfite Sequencing and Analysis**

Methylation patterns were experimentally validated via whole-genome bisulfite sequencing. DNA was extracted from a 2mg pool of flash frozen Insectary females using the QIAGEN® DNeasy Blood and Tissue Kit according to manufacturer’s instructions. Extracted DNA was suspended in TE buffer and quantified by Qubit (Thermo Fisher Scientific). Libraries were made with in-house Illumina-compatible protocol. The extracted DNA was fragmented by S-series focused ultrasonicator (Covaris) using the “200bp-target peak size protocol”. Fragmented DNA was then size selected (200bp-600bp) with Agencourt AMPure XP bead-based (Beckman Coulter Cat. No. A63880) size selection protocol [129]. The DNA End repair step was performed with End-It DNA end repair kit (Epicentre, Cat. No. ER81050). After end repair step, A-tailing (NEB, cat. No. M0202) and ligation steps were performed to ligate methylated adaptors. Bisulfite treatment of genomic DNA was performed using the MethylCode Bisulfite Conversion Kit (Life technologies). Purified genomic DNA was treated with CT conversion reagent in a thermocycler for 10 minutes at 98C, followed by 2.5 hours at 64C. Bisulfite-treated DNA fragments remain single-stranded as they are no longer complementary. Low-cycle (4-8) PCR amplification was performed with Kapa HiFi Uracil Hotstart polymerase enzyme (KAPA Biosystems, cat. No. KK2801), which can tolerate uracil residues. The final library fragments contain thymines and cytosines in place of the original unmethylated cytosine and methylated cytosines, respectively. The methylome libraries were diluted and loaded onto Illumina HiSeq 2500 system for sequencing using 150bp single-end reads. Reads were checked for quality with FastQC (http://www.bioinformatics.babraham.ac.uk/projects/fastqc/) and trimmed using Trim Galore! (http://www.bioinformatics.babraham.ac.uk/projects/trim_galore/). Genome indexing, alignment (Bowtie2 mapping parameters: --score_min L,0,-0.4), methylation calling and extraction were performed using Bismark [130]. Fractional CpG methylation of genes (log transformed) were plotted against gene CDS CpG O/E. See Table S2 for SRA information for the BisSeq reads.

**S3.4 Conservation of Methylation**

The same set of 1:1:1 orthologous genes used for protein rate testing was used to look at conservation of methylation across species. BS-seq data were obtained for *Apis mellifera* [131], *Nasonia vitripennis* [38], and *Trichogramma pretiosum*. To determine the status of methylation in each ortholog, we categorized genes with an average gene body methylation of > 0.01 as methylated genes and the rest as unmethylated genes.

**S4. COMPARISONS TO A SEXUAL *TRICHOGRAMMA PRETIOSUM***

**S4.1** **Biological Materials, Sequencing, and Assembly**

To identify which features of the *Trichogramma pretiosum* genome are unique to the asexual line, and which are features of *Trichogramma* evolution more generally, we obtained draft genome sequence for a sexual line of *Trichogramma pretiosum.* A single sib-mated female collected from Irvine, California in 2008 was used to initiate an inbred line “CA-29”, which was previously described [64]. *Wolbachia* infected *Trichogramma pretiosum* have not been collected in California before [19], so these wasps represent sexually reproducing populations naïve to *Wolbachia*. Infection status and species identification were confirmed using the same methods as for the Insectary line [5, 65-66]. Wasps are maintained in 12 x 75 mm glass culture tubes stopped with cotton and incubated at 24^o^C, L:D = 16:8, with 50% relative humidity. Every 10 days cultures are offered fresh honey and egg cards made of irradiated *Ephestia kuehniella* host eggs (Beneficial Insectary, Guelph, Ontario, Canada) adhered to card stock with double-sided tape.

Wasps were mass reared for several generations and collected into 100% ethanol, after which DNA was extracted with the QIAGEN® DNeasy Blood and Tissue Kit. 1 µg of the DNA was sheared using a Covaris S-2 system (Covaris, Inc. Woburn, MA) to 600bp and the NEBNext® Ultra™ DNA Library Prep Kit for Illumina® was used to make libraries for sequencing according to manufacturer’s instructions. Sequencing was performed on an Illumina® HiSeq2500 with RAPID mode to generate 2X250 reads. We generated 27,480,751 paired end, 250 base pair reads, amounting to ~70X coverage of the reference i5k genome for the asexual *Trichogramma pretiosum.* We attempted genome assembly of the CA-29 genome using a variety of programs and parameters. We used MaSuRCA v2.3.2 [67], SOAPdenovo2 v2.04 [68], and CLC Genomics Workbench v6.0.2 (CLC bio, Aaarhus, Denmark). Results are in Table S6.

Raw reads have been deposited in the NCBI Short Read Archive with the same project number as the reference: PRJNA168121 (SRP040113), sample CA-29 (SRS2813656), experiment SRX3538457, and run: SRR6447489.

**Table S6.** Sexual CA-29 *Trichogramma pretiosum* genome assembly results

| **Program** | **Parameters** | **Scaffold Count** | **Scaffold N50** | **Total Nucleotides** |
| --- | --- | --- | --- | --- |
| MaSuRCA | K-mer size = auto, k=127 chosen. Jellyfish hash = 13600000000, all other parameters as suggested for eukaryotic illumina only genomes. | 40,567 | 8,270 | 189,928,596 |
| SOAPdenovo2 | -K 35 through 127, k=127 had least fragmented assembly, results shown. | 134,158 | 9,895 | 198,666,185 |
| CLC Genomics Workbench | Reads cleaned with “Trim Sequences”, standard parameters. Automatic word size, bubble size 125, minimum contig length 500bp, auto-detect PE distance. | 24,015 | 15,741 | 177,355,685 |

**S4.2 Alignment and Comparison to the Reference**

We chose the MaSuRCA assembly to compare to the reference (Table S4), representing the best tradeoff between fragmentation, scaffold number, and length. We performed a whole genome alignment with the i5k “Insectary” genome as the reference, and the sexual “CA-29” genome as the query using MUMMER [69]. We used the wrapper DNADIFF to align the assemblies with NUCMER, identify SNPS and indels, and summarize the alignments. SNPs and indels were converted to VCF format and we used snpEff [70] to determine functional consequences and location of the variants using a custom built database of the i5k *Trichogramma pretiosum* “Insectary” genome.

**S4.3 Divergence (dN/dS) Estimates**

For dN/dS calculations, variants were substituted into the reference genome to build a reference for CA-29. VCFtools v.0.1.13 was used to write the SNPs only VCF [71], and GATK v.3.8 “FastaAlternateReferenceMaker” was used to substitute the variants [72]. CA-29 nucleotide fasta files were written with the cufflinks v.2.2.2 utility “gffread” [73], and trimmed to contain only the exon sequence with a custom perl script. Nucleotide alignments were created with MUSCLE v3.8.425 [74] based on back translated protein alignments (with reference to the unaligned nucleotide sequence) to preserve codon positions. Synonymous (dS) and nonsynonymous (dN) divergence was calculated with the maximum-likelihood based Yang-Nielson algorithm [75].

**S5. IMMUNITY**

**S5.1 Immune Gene Searches**

The set of genes differentially regulated upon bacterial infection in *Nasonia vitripennis* [45] were queried against the *Trichogramma pretiosum* coding-sequences using blastp [119], and an e-value cutoff of 1e-5 was used to determine presence or absence of a gene. Hits were categorized based on the taxonomic groupings set forth in [Sackton, et al. [](#_ENREF_52)45].

**S5.2 Immunity Results**

There are broad classes of immunity genes that are shared across insects. However, taxon specific immunity genes have received less attention. [Sackton, et al. [](#_ENREF_52)45] revealed a set of bacterial infection responsive genes in the parasitoid wasp *Nasonia vitripennis*. We therefore searched for orthologs of these in *Trichogramma pretiosum*. *Trichogramma pretiosum* has orthologs for 156 of the 244 *Nasonia vitripennis* immunity genes (Table 7). The larger the clade for which the gene is found to be specific (ex., “Metazoa” vs. “Insect”), the more likely a *Trichogramma pretiosum* ortholog was identified. The only exception to this trend was for “arthropod” immunity genes, but that was also the smallest category of genes (n = 6). Twenty percent of the genes identified as *Nasonia vitripennis*-specific were identified in *Trichogramma pretiosum*, indicating they are not specific to *Nasonia* as previously described. In addition to the lineage-specific nature of immunity genes, it is likely that *Trichogramma pretiosum* does not have a strong need for a diverse repertoire of immune genes, as it spends most of its life in a relatively sterile environment: the host insect egg. In comparison, *Nasonia* encounters a large diversity of microbes during development within the pupal stage of flies that have been feeding and living in septic environments.

**Table S7.** Numbers of infection-responsive genes from *Nasonia vitripennis* present in *Trichogramma* *pretiosum*.

| **Specificity^a^** | ***Nasonia vitripennis*** | ***Trichogramma pretiosum*** | **% Detected in *Trichogramma pretiosum*** |
| --- | --- | --- | --- |
| Metazoa | 102 | 91 | 89.2 |
| Arthropod | 6 | 6 | 100 |
| Insect | 49 | 39 | 79.6 |
| Hymenoptera | 14 | 6 | 42.9 |
| “Wasp” | 70 | 14 | 20.0 |
| Total | 244 | 156 | 63.9 |

^a^Defined by [Sackton, et al. [](#_ENREF_52)45]

**S6. REPETITIVE ELEMENTS**

**S6.1 K-mer Analysis to Estimate Repeat Compositions**

To estimate the repeat composition of the two strains of *Trichogramma pretiosum*, we used an alignment-free and sequence assembly-free k-mer counting approach. Jellyfish (http://www.genome.umd.edu/jellyfish.html) was used to count the number of occurrences of all 17-mers in the Illumina libraries for the sexual and asexual lines. For the asexual reference genome, the 500bp library was used for the following analyses. The modes of two k-mer frequency distributions, excluding k-mers that corresponded to sequencing errors, were 31X and 37X respectively for the sexual and asexual strains (Figure S4). These peaks, which correspond to single-copy portions of the genomes, amounted to the length of 143.2 Mb and 138.4 Mb of the sexual and asexual genomes, respectively. The estimated total genome sizes and the sizes of the repeated portions of the two genomes were found to be similar (Table S8). The complete distributions of 17-mers are provided in Figure S5.

**Figure S4.** Distribution of 100 least occurring 17-mers in the Illumina libraries of the sexual (**A**) and asexual (**B**) strains of *Trichogramma pretiosum*. Vertical lines represent the peak k-mer frequencies. Portions of the k-mer distribution that correspond to sequence-error-free 17-mers are highlighted by circles.

**Table S8.** K-mer based genome size estimates of the two sequenced strains of *Trichogramma* *pretiosum*

|  | **Total genome size (Mb)** | **Size (and %) of single copy portion of genome** | **Size (and percentage) of repeated portion of the genome** |
| --- | --- | --- | --- |
| Sexual | 193.9 | 143.2 (73.8 %) | 50.7 (26.2%) |
| Asexual | 199.0 | 138.7 (69.5 %) | 60.3 (30.3%) |

**Figure S5.** Distribution of all 17-mers in the Illumina libraries of the sexual (A) and asexual (B) lines of *Trichogramma pretiosum*. Vertical lines represent the peak k-mer frequencies.

**Table S9.** Annotations of transposable elements identified from Illumina reads obtained from sexual and asexual strains of *Trichogramma pretiosum*.

| **Repeat Class** | **Fragments (sexual)** | **Length (sexual)** | **Fragments (asexual)** | **Length (asexual)** |
| --- | --- | --- | --- | --- |
| Multicopy gene | 7 | 3796 | 4 | 406 |
| rRNA | 7 | 3796 | 4 | 406 |
| Simple Repeat | 3 | 769 | 6 | 1420 |
| Satellite | 3 | 769 | 6 | 1420 |
| MSAT | 1 | 240 | 1 | 219 |
| SAT | 2 | 529 | 5 | 1201 |
| Transposable Element | 126 | 69513 | 276 | 159025 |
| DNA transposon | 20 | 9577 | 60 | 28061 |
| EnSpm/CACTA | 4 | 1430 | 13 | 9160 |
| Ginger2/TDD | 0 | 0 | 2 | 373 |
| Helitron | 3 | 1232 | 3 | 1427 |
| ISL2EU | 1 | 735 | 6 | 2451 |
| Kolobok | 0 | 0 | 2 | 483 |
| Mariner/Tc1 | 4 | 1960 | 8 | 3478 |
| MuDR | 0 | 0 | 7 | 4295 |
| P | 0 | 0 | 8 | 3180 |
| Transib | 0 | 0 | 8 | 2113 |
| MuDR | 5 | 3347 | 0 |  |
| Polinton | 2 | 666 | 0 |  |
| hAT | 1 | 207 | 3 | 1101 |
| LTR Retrotransposon | 67 | 37118 | 161 | 98261 |
| BEL | 17 | 11185 | 22 | 13464 |
| Copia | 0 | 0 | 14 | 11292 |
| DIRS | 0 | 0 | 4 | 5300 |
| Gypsy | 50 | 25933 | 121 | 68205 |
| Non-LTR Retrotransposon | 39 | 22818 | 55 | 32703 |
| CR1 | 8 | 4209 | 9 | 3959 |
| I | 2 | 1242 | 9 | 6066 |
| Jockey | 1 | 624 | 4 | 2155 |
| Kiri | 1 | 480 | 0 |  |
| L2 | 1 | 636 | 2 | 525 |
| Nimb | 0 | 0 | 1 | 1008 |
| Penelope | 2 | 1266 | 3 | 1536 |
| R1 | 19 | 10899 | 21 | 13504 |
| R2 | 5 | 3462 | 6 | 3950 |
| **Total annotated** | **136** | **74078** | **286** | **160851** |

**S6.2 K-mer Based Inference of Transposable Elements**

We also implemented an alignment and assembly based approach to estimate the transposable element composition of the genomes of the two strains. We used Tedna [41] to infer transposable elements from Illumina libraries (‘read sets’) of the two strains. Tedna assembles transposable elements (TE) by assembling a set of highly repeated long k-mers, using a de Bruijn graph. From each read set, we first extracted 5 millions reads, chosen at random, and implemented tedna with the default k-mer size of 61 (parameter -k). We inferred 454 and 231 TEs from the asexual and the sexual strain’s read sets, respectively. These TE libraries were annotated using CENSOR, using the ‘translated search’ option and the ‘sequence source’ option set to ‘Arthropoda’ [42] (Table S9). In order to estimate the relative contribution of these TEs to the genomes of these strains, we mapped both the read sets to the both the TE libraries using ‘bwa-mem’ with default options, and measured the number of read that mapped to the TE libraries using samtool’s flagstat tool [43-44]. In order to estimate the relative contribution of these TEs to the genomes of these strains, we mapped both the read sets to the both the TE libraries using ‘bwa-mem’ with default options after masking microsatellite repeats by identifying their coordinates using Tandem Repeat Finder (TRF) [81], and measured the number of read that mapped to the TE libraries using samtool’s flagstat tool [43,44]. A greater percentage of genomic reads from sexual strain mapped to both the TE libraries as compared to asexual strain, suggesting genomic enrichment of repeats in the sexual strain (**Table S10**).

**Table S10.** Number and percentage of total genomic reads from sexual and asexual strains that mapped separately to *tedna*-derived repeat libraries from sexual and asexual strains.

|  | **Reads (Sexual) mapped to TE library from Sexual strain** | **Reads (Asexual) mapped to TE library from Sexual strain** | **Reads (Sexual) mapped to TE library from Asexual strain** | **Reads (Aexual) mapped to TE library from Aexual strain** |
| --- | --- | --- | --- | --- |
| **Total number of reads** | 55,195,406 | 142,398,418 | 55,195,406 | 142,398,418 |
| **No. reads mapped** | 6,244,171 | 11,005,393 | 7,065,452 | 12,055,826 |
| **Percentage of total reads mapped to TE library** | 11.31% | 7.73% | 12.77% | 8.47% |
| **No. properly mapped read pairs** | 5,791,220 | 8,604,762 | 6,402,028 | 8,311,664 |
| **Percentage of properly mapped read pairs** | 10.54% | 6.05% | 11.65% | 5.85% |

Many of the putative transposable elements contain short and long tandem repeat sequences. We sought to estimate whether the differential mapping of genomic reads from the two strains to the tedna TE libraries is a consequence of differences in expansion of transposon elements or due to changes in long tandem arrays in the genomes. In the tedna TE library, we identified the coordinates of long tandem arrays (motif size > 10) identified by TRF (‘tandem array portions’), and the coordinates corresponding to transposon annotations by CENSOR (‘transposon portions’). We removed any transposon portions that overlapped with tandem arrays, and then counted the number of reads that mapped to each of these partitions. To ensure that only repeated sequences are being compared, we removed any regions that had less than 500 reads mapping to them in either of the strains. We then identified those transposon regions in which either sexual or asexual strain had more than 5 times the coverage of the other strain, controlling for the differences in the genomic library sizes of these two strains. We did not identify any transposon region that has evidence of expansion in the asexual strain. However, we identified multiple LTR-like elements that show evidence of expansion in sexual strain. (Table S11). However, the difference in read coverage of these two strains for these individual repeats (e.g., difference of 8731 reads for *Chimpo_I*) does not explain the large difference in the number of reads mapped to the tedna-derived libraries between the two strains (Table S10). We identified multiple long tandem arrays that showed greater differences in read coverage between the two strains (e.g., 78051 for a 63-bp long tandem array) (Table S12), suggesting that the genome of the sexual strain may have experienced expansion of tandem repeats. Large numbers of the longer (>100 bp) repeats identified are themselves composed of smaller repeats (<35 bp), some of which can be further decomposed into even smaller units (<15 bp). It should be noted that these results could differ from approaches that use alignment based methods for identifying repeat elements.

**Table S11.** Transposon-like elements with differential read coverage in sexual and asexual strains

| **Repeat Name** | **Repeat Class** | **No. reads mapped in sexual strain** | **No. reads mapped in asexual strain** | **Evidence of expansion in** |
| --- | --- | --- | --- | --- |
| Chimpo_I | LTR/Gypsy | 13091 | 4360 | Sexual strain |
| BEL-3-I_NVi | LTR/BEL | 2551 | 803 | Sexual strain |
| BEL-3-I_NVi | LTR/BEL | 2412 | 807 | Sexual strain |
| BEL-9_LH-I | LTR/BEL | 1827 | 644 | Sexual strain |
| Gypsy-3_RP-I | LTR/Gypsy | 1792 | 647 | Sexual strain |
| BEL1-NVi_I | LTR/BEL | 2184 | 822 | Sexual strain |
| Rehavkus-2_NVi | DNA/MuDR | 1036 | 525 | Sexual strain |
| BEL-2-I_NVi | LTR/BEL | 2087 | 965 | Sexual strain |
| BEL-3_LH-I | LTR/BEL | 2063 | 952 | Sexual strain |
| Gypsy1-NVi_I | LTR/Gypsy | 1777 | 686 | Sexual strain |

**Table S12.** Results of mapping genomic reads from the two strains to long tandem array sequences identified by Tandem Repeat Finder within tedna repeat libraries

| **Repeating motif** | **Motif length (bp)** | **Identified in tedna library derived from** | **No. reads mapped in sexual** | **No. reads mapped in asexual** |
| --- | --- | --- | --- | --- |
| ATATAGAAGTAAAAGAGAGAGAGAGAAAACAAGCGAGAAGAACCCAAGCTATCGAGACACGGT | 63 | Sexual strain | 37043 | 13956 |
| CAACTCGGCAACGATAAAATGCGGCCTACGAAGAATGACACTCTCGTCTTAGCCAAAAGCACGAGAAGCGATAACAGTCTCCTCTACC | 88 | Sexual strain | 24619 | 12386 |
| AACGCTCAGT | 10 | Sexual strain | 7898 | 3628 |
| GACAATGAAATCATCCATGAAATAAGTTTG | 30 | Asexual strain | 3743 | 1488 |
| CACGCACTCTTGCACGTGCA | 21 | Asexual strain | 46561 | 9473 |
| TCTCTCTCTCTCTCTTTTACTTCTATATACAGTGTCACGATAGCTTGGGTTCTCCGCACTCGTTTTCTTTCATTCTCTTTTACTCCTATATACCGCGTCTCAATACCCAAGGTTCTCTACACTCGTTTTC | 130 | Asexual strain | 84226 | 6175 |
| TCACTCAACGTTGACGCACAATAACGTACGCTAACGAACTTCAACGCTCAATAACGCTATGTAACGCGCAGTAACGCCCTCTACTACACGTAACTGATCGTGACTTAACGTTACTGCACATGACTGCACGTTATTGAA | 138 | Asexual strain | 29622 | 12633 |
| TTAGTCGAGATAACAGAAGTCGATGAGAACATTGACATTTTTCTGGACAAAACTGAGGACGCTACGCATACACAGGCAATAACGGCAGGAGCCCGAGAATCTCCTACTTCTGTATAAGATTTTGGTATAACTTTGTACGCTAACCGACCAAGTTCGAGAAACTAGTATCTCAAACACGGTAGCTCACGTTTCATACACTCGATGACTTGACGAGACATAAACGTCCAACAAGACATTGCTTAGGTTTCAAGCTGGTGCTGTTGTTTTGTCTTATATTTCTATAGGCGTTTAGTCGAGATAACAGAAGTCGATGAGAACA | 319 | Asexual strain | 21550 | 7861 |
| TTAGTCGAGATAACAGAAGTCGATGAGAACA | 31 | Asexual strain | 13166 | 2096 |
| CACATGCATGTGTGACACCCATGTGCACTACATCGATGTGGGTTTTGAGATAACCCTTTGTTGAAAATTTCTTTCCGCAAATAT | 81 | Asexual strain | 16538 | 7708 |
